# Hepatitis C Virus Subverts Human Choline Kinase-α To Bridge Phosphatidylinositol-4-Kinase IIIα (PI4KIIIα) and NS5A and Upregulates PI4KIIIα Activation, Thereby Promoting the Translocation of the Ternary Complex to the Endoplasmic Reticulum for Viral Replication

**DOI:** 10.1128/JVI.00355-17

**Published:** 2017-07-27

**Authors:** Mun-Teng Wong, Steve S. Chen

**Affiliations:** Institute of Biomedical Sciences, Academia Sinica, Taipei, Taiwan, Republic of China; Hudson Institute of Medical Research

**Keywords:** HCV, NS5A, hCKα, PI4KIIIα, PI4P, ternary complex, ER translocation

## Abstract

In this study, we elucidated the mechanism by which human choline kinase-α (hCKα) interacts with nonstructural protein 5A (NS5A) and phosphatidylinositol-4-kinase IIIα (PI4KIIIα), the lipid kinase crucial for maintaining the integrity of virus-induced membranous webs, and modulates hepatitis C virus (HCV) replication. hCKα activity positively modulated phosphatidylinositol-4-phosphate (PI4P) levels in HCV-expressing cells, and hCKα-mediated PI4P accumulation was abolished by AL-9, a PI4KIIIα-specific inhibitor. hCKα colocalized with NS5A and PI4KIIIα or PI4P; NS5A expression increased hCKα and PI4KIIIα colocalization; and hCKα formed a ternary complex with PI4KIIIα and NS5A, supporting the functional interplay of hCKα with PI4KIIIα and NS5A. PI4KIIIα inactivation by AL-9 or hCKα inactivation by CK37, a specific hCKα inhibitor, impaired the endoplasmic reticulum (ER) localization and colocalization of these three molecules. Interestingly, hCKα knockdown or inactivation inhibited PI4KIIIα-NS5A binding. In an *in vitro* PI4KIIIα activity assay, hCKα activity slightly increased PI4KIIIα basal activity but greatly augmented NS5A-induced PI4KIIIα activity, supporting the essential role of ternary complex formation in robust PI4KIIIα activation. Concurring with the upregulation of PI4P production and viral replication, overexpression of active hCKα-R (but not the D288A mutant) restored PI4KIIIα and NS5A translocation to the ER in hCKα stable knockdown cells. Furthermore, active PI4KIIIα overexpression restored PI4P production, PI4KIIIα and NS5A translocation to the ER, and viral replication in CK37-treated cells. Based on our results, hCKα functions as an indispensable regulator that bridges PI4KIIIα and NS5A and potentiates NS5A-stimulated PI4KIIIα activity, which then facilitates the targeting of the ternary complex to the ER for viral replication.

**IMPORTANCE** The mechanisms by which hCKα activity modulates the transport of the hCKα-NS5A complex to the ER are not understood. In the present study, we investigated how hCKα interacts with PI4KIIIα (a key element that maintains the integrity of the “membranous web” structure) and NS5A to regulate viral replication. We demonstrated that HCV hijacks hCKα to bridge PI4KIIIα and NS5A, forming a ternary complex, which then stimulates PI4KIIIα activity to produce PI4P. Pronounced PI4P synthesis then redirects the translocation of the ternary complex to the ER-derived, PI4P-enriched membrane for assembly of the viral replication complex and viral replication. Our study provides novel insights into the indispensable modulatory role of hCKα in the recruitment of PI4KIIIα to NS5A and in NS5A-stimulated PI4P production and reveals a new perspective for understanding the impact of profound PI4KIIIα activation on the targeting of PI4KIIIα and NS5A to the PI4P-enriched membrane for viral replication complex formation.

## INTRODUCTION

Like many other positive-sense RNA viruses, the hepatitis C virus (HCV) exploits cellular lipids and reorganizes endoplasmic membranes to create a specialized viral replication membrane compartment, which is referred to as the “membranous web” (MW) (1, 2). The MW serves as a compartmentation and concentration site for the HCV replication complex (RC), which is composed of the NS3 (nonstructural protein 3) to NS5B proteins, viral replicating RNA, and a wide array of host factors (3, 4). The MW is an endoplasmic reticulum (ER)-derived membrane structure, highly phosphorylated and remodeled, that has heterogeneous vesicles ranging from 100 to 300 nm in diameter and is dedicated to HCV replication (5).

Phosphatidylinositol-4-kinases (PI4Ks) catalyze the phosphorylation of position 4 of the inositol ring in phosphatidylinositol (PI) to form PI-4-phosphate (PI4P) and play a crucial role in the regulation of the intracellular membrane trafficking process (6, 7). PI4P is an essential precursor in the synthesis of PI(4,5)P2 and PI(3,4,5)P3, which are necessary for receptor-activated phospholipase C and phosphoinositide 3-kinase signaling (8), and controls cellular trafficking, especially in the Golgi apparatus and trans-Golgi network (8). PI4Ks exhibit distinct subcellular localization and affect the compartmentation of discrete PI4P pools. PI4KIIIα localizes primarily to the ER and maintains a distinct population of PI4P that is critical for ER export when cells are responding to the increased ER cargo load (9, 10). PI4KIIIα also regulates the PI4P level at the plasma membrane (11, 12). PI4KIIIα can also be found in close association with the membranes of the Golgi vesicles and vacuoles (13) and in the nucleolus (14). PI4KIIIβ is the lipid kinase primarily responsible for the PI4P pool in the Golgi apparatus (15, 16), and it also localizes on the lysosome to maintain lysosomal membrane integrity (17).

PI4K isoforms and their product PI4Ps play crucial roles in the replication of many positive-stranded RNA viruses in the Flaviviridae, Picornaviridae, and Coronaviridae families, shaping the architecture of their membranous replication compartments via the elevation of PI4P production (7). PI4KIIIα has been identified as a crucial host factor in HCV replication (18–23). Although PI4KIIIβ has been reported to play a role in the viral replication of genotypes 1a and 1b (19, 22), its role in genotype 2a replication is less clear (18, 24).

As a critical component of the viral RC, membrane-associated NS5A is present in both a hypophosphorylated p56 form and a hyperphosphorylated p58 form (25, 26), which modulate HCV RNA replication and virus assembly (27). NS5A interacts intimately with PI4KIIIα via the C-terminal part of domain 1 and recruits the kinase to the viral RC site, where NS5A stimulates kinase activity in conjunction with NS5B, thereby leading to the generation of elevated PI4P pools at intracellular membranes (24, 28). PI4KIIIα activation and PI4P redistribution are required for the changes in the ultrastructural architecture of HCV RCs embedded within the MW (18, 21, 24, 29). Analogously to the PI4KIIIα depletion phenotype, mutations in the seven amino acids within NS5A, which have been identified as a PI4KIIIα functional interaction site (PFIS), not only interfere with PI4KIIIα binding and attenuate PI4P production but also alter the morphology of viral replication sites (29). Conversely, PI4KIIIα controls HCV RNA replication, presumably via alteration of the phosphorylation status of NS5A (29). Thus, NS5A interplays with PI4KIIIα to promote PI4P production and endoplasmic membrane reorganization, which are necessary for viral replication. In addition, the accumulation of PI4P recruits lipid transport proteins, such as oxysterol-binding protein (30) or the four-phosphate adaptor protein (31), which regulate the trafficking of cholesterol and glycosphingolipids, respectively, to the viral MW for effective viral genome replication.

Recently, Harak et al. demonstrated that HCV depends on optimal levels of PI4KIIIα activity for proper replication and showed that the replication of various genotypes requires PI4KIIIα activation at a low PI4KIIIα protein level, such as that observed for primary hepatocytes (32). Nevertheless, in cultured hepatoma Huh7 cells, which already express higher levels of PI4KIIIα than primary hepatocytes, NS5A-induced PI4KIIIα activation leads to excess PI4P production that is harmful to their replication (32). To manage the growth disadvantage in Huh7 cells, these isolates have evolved a mechanism through adaptive mutations, such as S2204R in NS5A and R2884G in NS5B, to limit excess PI4KIIIα activation, thus ensuring their proper replication in Huh7 cells (32). Strikingly, the genotype 2a JFH1 virus is an exception, because it does not require adaptive mutations for robust replication in Huh7 cells (33). Thus, PI4KIIIα activity delicately adapts to the viral and cellular microenvironment to ensure efficient viral replication. Nevertheless, whether the NS5A-mediated recruitment and activation of PI4KIIIα require a yet-to-be identified cellular factor has not been clarified, and the mechanism underlying the regulation of PI4KIIIα and NS5A trafficking to the PI4P-enriched viral replication site has not been identified.

Choline kinase (CK), the first enzyme in the CDP-choline (or Kennedy) pathway, catalyzes the phosphorylation of choline into phosphocholine. Previous genome-wide small interfering RNA (siRNA) screens have implicated human CKα (hCKα) in HCV infections (24, 34). As we demonstrated previously, hCKα activity enhances the translocation of the hCKα-NS5A complex to the ER, where the hCKα protein mediates the NS5A-NS5B interaction, thereby promoting functional membranous viral RC assembly and viral RNA replication (35). However, whether hCKα interacts with PI4KIIIα and NS5A to modulate the viral replication process has not been clarified. In addition, the mechanism underlying the regulation of hCKα-mediated translocation of the hCKα-NS5A complex to the ER remains to be elucidated.

Because PI4K isoforms play critical roles in intracellular membrane trafficking (6, 36) and PI4KIIIα builds a PI4P-enriched membrane platform for effective HCV replication (37, 38), we hypothesize that the hCKα activity-mediated ER translocation of hCKα and NS5A is tightly associated with PI4KIIIα activation and that hCKα activity plays a crucial role in NS5A-stimulated PI4KIIIα activation and in the translocation of PI4KIIIα and NS5A to the ER, thereby promoting membranous viral RC assembly and viral replication (35).

In the present study, we demonstrate that HCV usurps hCKα functions to facilitate viral replication. hCKα acts as a novel upstream adaptor to bridge PI4KIIIα and NS5A, thereby forming a PI4KIIIα ternary complex. Additionally, the results obtained from a cell culture system and an *in vitro* kinase assay demonstrate that hCKα activity slightly enhances PI4KIIIα basal activity but greatly potentiates NS5A-stimulated PI4KIIIα activity, indicating that hCKα synergistically upregulates PI4KIIIα activity in conjunction with NS5A. In turn, the massive production of PI4P pools facilitates the translocation of the ternary complex to the ER-derived, PI4P-enriched membrane for viral replication. Our study highlights the novel viewpoint that PI4KIIIα activity and its translocation in conjunction with NS5A to viral replication sites are regulated by the cellular kinase hCKα, even though the product of this kinase is not directly linked to membrane composition or structure.

## RESULTS

### hCKα colocalizes with NS5A and PI4P or PI4KIIIα in HCV-expressing cells.

We recently demonstrated that hCKα activity is critical for the intracellular trafficking of the hCKα-NS5A complex to the ER membrane, where hCKα mediates the binding of NS5A to NS5B for viral RC assembly (35). In the present study, we further examined whether hCKα interacts with PI4KIIIα, the lipid kinase critical for the integrity of the HCV-induced MW, and NS5A to stimulate PI4P production and whether accumulated PI4P production facilitates the translocation of hCKα and NS5A, as well as PI4KIIIα, to the ER membrane, thereby promoting HCV replication.

**FIG 1:**
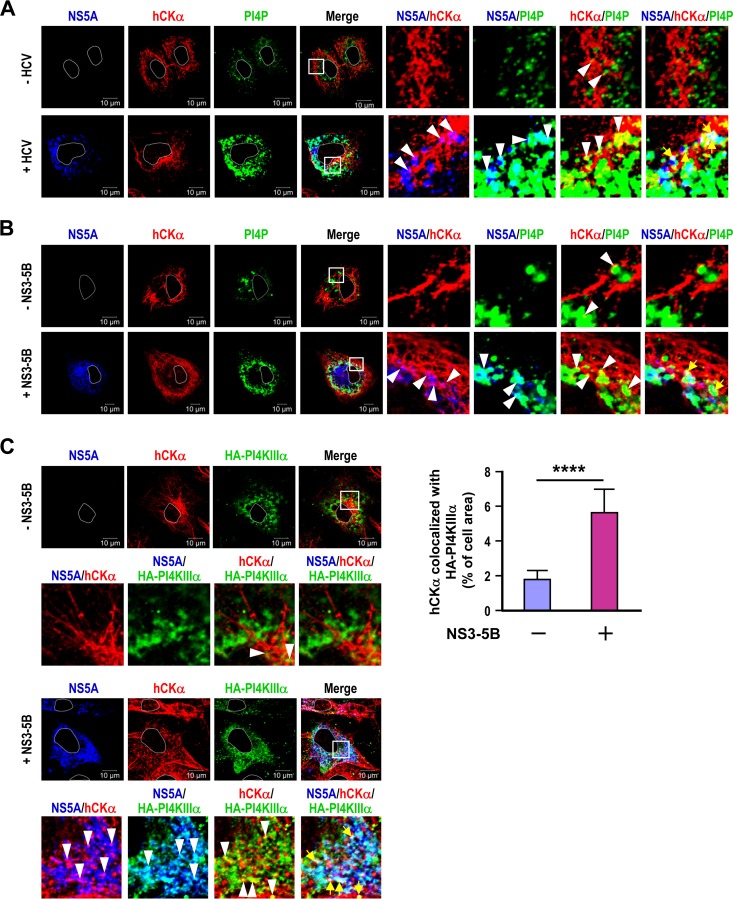
Colocalization of hCKα with NS5A and PI4P or PI4KIIIα. (A) Huh7 cells either remained uninfected or were infected with HCVcc at an MOI of 1. At day 3 postinfection, the cells were fixed and successively immunostained with rabbit anti-hCKα, a mouse anti-NS5A (9E10) MAb (IgG), and mouse anti-PI4P (IgM), followed by incubation with appropriate Alexa Fluor dye-conjugated secondary antibodies. The cells were then analyzed by confocal microscopy. Nuclei are demarcated by solid white lines. The images of the three fluorescence signals were merged, and the boxed areas were enlarged. The colocalization of two or three molecules as indicated in the fluorescence profiles was marked by white arrowheads or yellow arrows, respectively. (B) T7/Huh7 cells were cotransfected with or without pTM-NS3–5B, and the cells were analyzed by confocal microscopy as described for panel A. (C) T7/Huh7 cells were transfected with pTM-HA-PI4KIIIα in the presence or absence of pTM-NS3–5B. The transfected cells were processed for confocal microscopy using rabbit anti-hCKα, MAb 9E10, and goat anti-HA. (Left) Fluorescence profiles were analyzed as described for panel A. (Right) The colocalization of hCKα with HA-PI4KIIIα in the presence or absence of NS3–5B expression was quantitated. The statistical significance of the values obtained in different experimental settings was calculated by applying the two-tailed, unpaired Student *t* test, and a *P* value of <0.05 was considered statistically significant. ****, *P* < 0.0001.

To address this issue, we first searched for the intracellular colocalization of hCKα, NS5A, and PI4P in HCV-infected Huh7 cells via confocal microscopy. As shown previously (35), a small fraction of hCKα colocalized with NS5A in HCV-infected cells (Fig. 1A, magenta puncta in the fluorescence image labeled “NS5A/hCKα”). HCV infection also resulted in much more accumulation of PI4P in dot-like patterns scattered in the cytoplasm than that seen with mock infection, and a fraction of these PI4P puncta colocalized with NS5A (Fig. 1A), which represents viral replication sites, in HCV-infected cells (Fig. 1A, cyan puncta in the image marked “NS5A/PI4P”). Interestingly, a small proportion of hCKα colocalized with PI4P in mock-infected cells, whereas HCV infection augmented hCKα and PI4P colocalization, as evidenced by the increased accumulation of yellow puncta in infected cells (Fig. 1A, image labeled “hCKα/PI4P”). Remarkably, a fraction of hCKα colocalized with NS5A and PI4P in HCV-infected cells (Fig. 1A, white puncta in the image marked “NS5A/hCKα/PI4P”).

Next, we employed the NS3–5B expression system, which produces proteins NS3 to NS5B independently of viral RNA replication, to investigate the intracellular localization of hCKα with NS5A and PI4P. PI4P localizes primarily on the Golgi apparatus in cells (28, 29). In NS3–5B-expressing cells, NS5A appeared as puncta dispersed in the cytoplasm (Fig. 1B). The expression of NS3–5B also resulted in higher levels of PI4P in the cytoplasm than in mock-transfected cells (Fig. 1B). As reported previously (28, 29), the PI4P puncta partially colocalized with NS5A (Fig. 1B, cyan puncta in cells expressing NS3 to NS5B). Strikingly, NS3–5B expression upregulated the colocalization of hCKα and PI4P, which was indicated by the yellow puncta present in NS3–5B-expressing cells (Fig. 1B).

To understand the involvement of hCKα in PI4KIIIα-catalyzed PI4P production, we then explored whether hCKα colocalized with NS5A and PI4KIIIα in T7/Huh7 cells that overexpressed hemagglutinin (HA)-PI4KIIIα, i.e., PI4KIIIα attached with an HA tag at its N terminus, with or without NS3–5B coexpression. The overexpressed HA-PI4KIIIα displayed dot-like structures scattered in the cytoplasm of cells expressing proteins NS3 to NS5B (Fig. 1C, left), and certain HA-PI4KIIIα puncta colocalized with those of NS5A (Fig. 1C, left). This observation was consistent with the current model in which PI4KIIIα interacts with NS5A and is recruited to viral replication sites (24). A small amount of hCKα colocalized with HA-PI4KIIIα in the absence of NS3–5B expression, whereas the expression of proteins NS3 to NS5B increased the colocalization of these two kinases (Fig. 1C, left). The percentage of the cell area that harbored fluorescent signals for both hCKα and HA-PI4KIIIα with or without viral NS protein expression was quantitated as described previously (35, 39). The results showed that the expression of NS3 to NS5B statistically upregulated the colocalization of hCKα with HA-PI4KIIIα (Fig. 1C, right). Additionally, hCKα colocalized with NS5A and PI4KIIIα in NS3–5B-expressing cells (Fig. 1C, left). Therefore, the results from Fig. 1 suggest that hCKα may impact viral replication through its involvement in NS5A-stimulated PI4KIIIα activation in HCV-expressing cells.

### NS5A-mediated PI4P accumulation requires hCKα activity.

**FIG 2:**
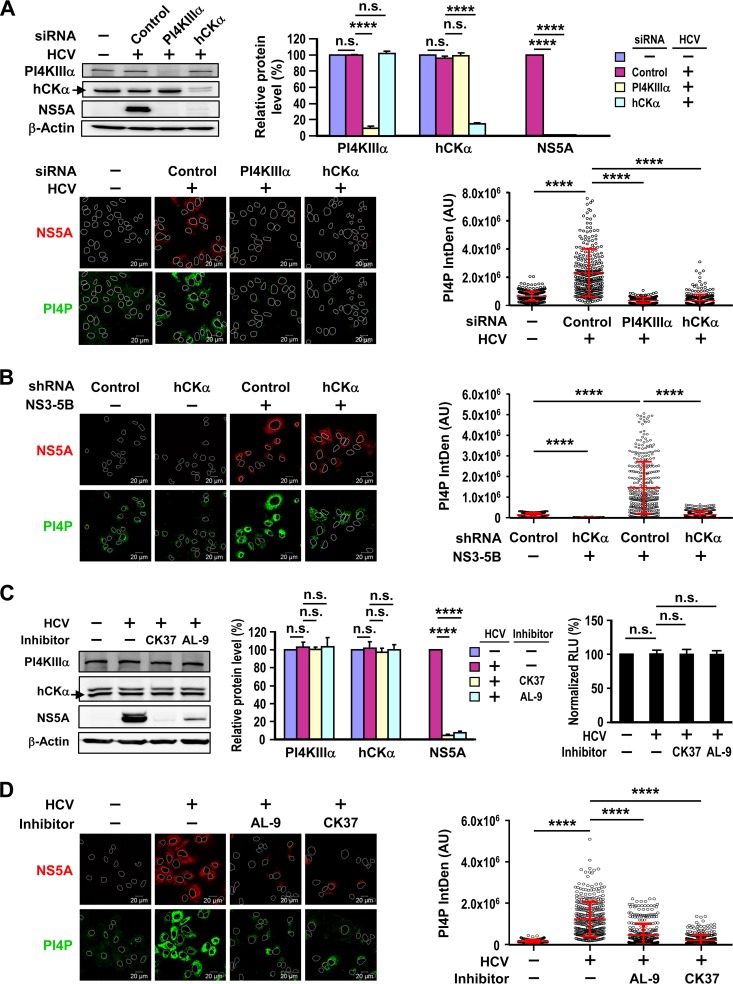
Effects of hCKα on HCV-mediated PI4P production. (A) (Top left) Huh7 cells were first transfected with nontargeted siRNA (control) or with PI4KIIIα or hCKα siRNA. The cells were then either mock infected (−) or infected with HCVcc at an MOI of 1. A subset of cells was subjected to Western blot analysis. The arrow marks the migration of hCKα in the immunoblot. (Top right) PI4KIIIα and hCKα levels were first normalized to those of β-actin, and these levels in different settings were expressed as percentages relative to the levels detected in mock-infected cells, which were arbitrarily assigned the value of 100%. NS5A levels, after normalization to β-actin levels, were detected in HCV-infected, siRNA-transfected cells and were compared to levels detected in HCV-infected cells transfected with control siRNA, which were arbitrarily assigned the value of 100%. ****, *P* < 0.0001; n.s., not statistically significant. (Bottom left) Another set of cells was processed by confocal microscopy using MAb 9E10 and anti-PI4P (IgM) to visualize the intracellular localization of NS5A and PI4P. A representative set of PI4P distribution patterns under different conditions is shown. (Bottom right) PI4P levels were quantitated as described in Materials and Methods. In each group, the PI4P fluorescence intensity was expressed as mean arbitrary units (AU) ± SD from 300 cells per condition. (B) Paired T7-control shRNA/Huh7 and T7-hCKα shRNA/Huh7 cells were transfected with the vector plasmid (−) or pTM-NS3–5B; the cells were monitored; and PI4P levels were quantitated. (C) (Left) Huh7 cells either remained mock infected or were infected with HCVcc, followed by treatment with DMSO as a vehicle (−), 100 μM CK37, or 5 μM AL-9. A set of cells was then analyzed by immunoblotting. (Center) Relative levels of the indicated cellular and viral proteins were quantified as described for panel A. (Right) Another set of cells was analyzed in a cell viability assay. Cell viability under different conditions was expressed as a percentage relative to that of mock-infected cells treated with the vehicle, which was arbitrarily designated 100%. (D) Huh7 cells either remained mock infected or were infected with HCVcc, followed by treatment with DMSO as a vehicle, AL-9, or CK37. The cells were then monitored, and PI4P levels were quantitated.

To explore whether hCKα contributes to PI4P production, we individually silenced hCKα and PI4KIIIα and compared the effects on PI4P levels in HCV-infected cells. PI4P levels were quantified as described previously (24, 29). As shown in Fig. 2A (top), HCV infection did not alter PI4KIIIα and hCKα levels. Individual transfection with PI4KIIIα and hCKα siRNAs knocked down PI4KIIIα and hCKα levels to approximately 9% and 15% of those detected with control siRNA transfection; these reductions occurred concomitantly with reduced NS5A expression (Fig. 2A, top). As shown in Fig. 1A, HCV infection augmented intracellular PI4P levels, and HCV-induced PI4P accumulation was ablated by PI4KIIIα or hCKα knockdown (Fig. 2A, bottom). These observations suggest a role for hCKα in PI4P production during HCV replication.

Next, we confirmed the inhibitory effects of stable hCKα knockdown on PI4P production in cells with or without NS3–5B expression. In the absence of NS3–5B expression, PI4P levels were lower in hCKα stable knockdown cells than in control knockdown cells (Fig. 2B). As shown in Fig. 1B, NS3–5B expression upregulated PI4P production in control stable knockdown cells (Fig. 2B). Interestingly, stable depletion of hCKα in NS3–5B-expressing cells attenuated the increase in PI4P production induced by NS3–5B expression (Fig. 2B). Together, these results imply that hCKα participates in PI4P production in cells regardless of NS3–5B protein expression, and hCKα may function upstream of PI4KIIIα-catalyzed PI4P production.

CK37 is a computationally identified compound that specifically inhibits hCKα by targeting its choline-binding site, leading to the inhibition of enzymatic activity (40). We showed previously that CK37 impairs viral RNA replication as well as viral protein expression in a dose-dependent manner (35). We then compared in parallel the inhibitory effects of CK37 and AL-9 on viral protein expression in HCV-infected cells. AL-9 is a prototypical PI4KIIIα inhibitor derived from 4-aminoquinazoline that targets PI4KIIIα activity and impairs viral replication by depleting PI4P in the plasma membrane (41). CK37 at 100 μM and AL-9 at 5 μM greatly decreased NS5A expression but did not affect PI4KIIIα and hCKα levels (Fig. 2C, left and center). Additionally, these two inhibitors did not influence the viability of HCV-infected cells (Fig. 2C, right).

To determine whether hCKα activity was critical for PI4P accumulation during HCV infection, we measured PI4P levels in mock-infected and HCV-infected cells treated with a vehicle, AL-9, or CK37. As expected, AL-9 greatly reduced HCV-induced PI4P accumulation (Fig. 2D), which is consistent with previous findings that PI4KIIIα is primarily responsible for PI4P production in HCV-expressing cells (24, 41, 42). Similarly, CK37 also impaired PI4P accumulation in HCV-infected cells (Fig. 2D), suggesting that hCKα activity promotes a functional interaction with PI4KIIIα and NS5A to regulate massive PI4P generation in HCV-expressing cells.

### hCKα activity upregulates NS5A-mediated PI4P production by activating PI4KIIIα.

**FIG 3:**
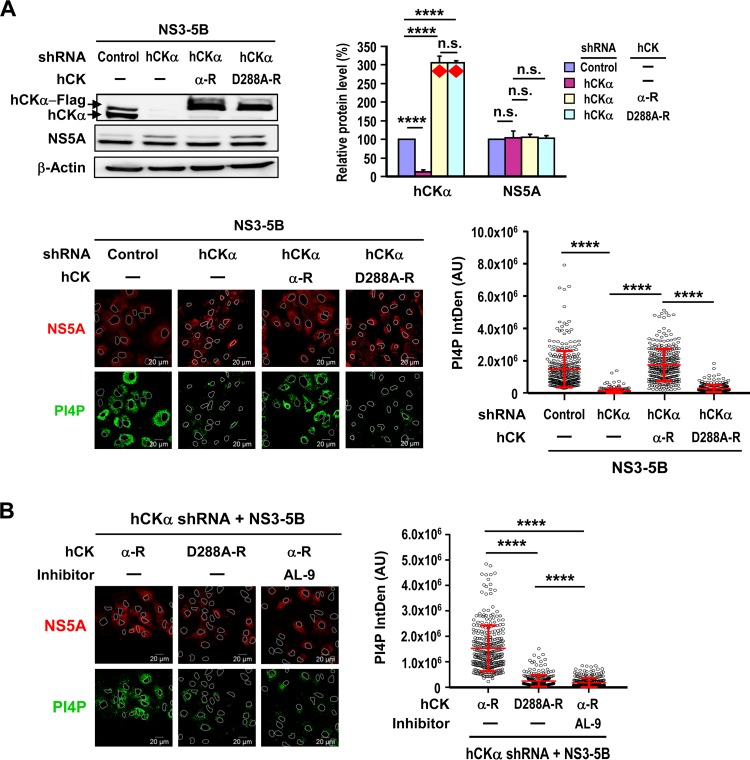
(A) (Top left) T7-control shRNA/Huh7 and T7-hCKα shRNA/Huh7 cells were cotransfected with pTM-NS3–5B and either a control plasmid (−), wild-type hCKα-R, or D288A mutant hCKα-R. The cells were then subjected to Western blot analysis. (Top right) Relative protein levels were quantified. Red diamonds mark the levels of overexpressed wild-type or D288A mutant hCKα-R relative to endogenous hCKα levels in control stable knockdown cells. Overexpressed and endogenous hCKα was detected with rabbit anti-hCKα in an immunoblot analysis. (Bottom) Another set of cells was monitored, and PI4P levels were quantified. (B) (Left) T7-hCKα shRNA/Huh7 cells were cotransfected with pTM-NS3–5B and wild-type or D288A mutant hCKα-R and were treated with a vehicle (−) or AL-9 as indicated above the images. (Right) The cells were then quantitated to determine PI4P levels. ****, *P* < 0.0001; n.s., nonsignificant.

To demonstrate the direct contribution of hCKα activity to elevated PI4P production, we evaluated the abilities of overexpressed wild-type hCKα-R and an inactive D288A mutant hCKα-R (35) to upregulate PI4P production in hCKα stable knockdown cells expressing proteins NS3 to NS5B. These two hCKα-R clones express authentic wild-type and mutant hCKα molecules whose mRNAs are resistant to the restrictive effects exerted by the hCKα short hairpin RNA (shRNA) used to construct hCKα stable knockdown cells (35). Stable knockdown of hCKα decreased hCKα levels by 87% from those detected in control stable knockdown cells (Fig. 3A, top). The levels of both wild-type and D288A mutant hCKα-R proteins overexpressed in hCKα stable knockdown cells were approximately 3-fold higher than the endogenous hCKα levels detected in control stable knockdown cells (Fig. 3A, top). Interestingly, overexpression of the wild-type, but not the D288A mutant, hCKα-R upregulated PI4P levels in hCKα stable knockdown cells relative to hCKα stable knockdown cells transfected with the vector control plasmid, and these levels were comparable to those detected in control stable knockdown cells (Fig. 3A, bottom).

To determine whether hCKα-regulated PI4P accumulation occurs via PI4KIIIα activation in HCV-expressing cells, the inhibitory effect of AL-9 on wild-type hCKα-mediated PI4P accumulation in hCKα stable knockdown cells was examined. Active hCKα-mediated PI4P production was greatly diminished by AL-9 to a level even lower than that detected in hCKα stable knockdown cells overexpressing D288A mutant hCKα-R (Fig. 3B). The residual PI4P levels observed in hCKα stable knockdown cells overexpressing mutant hCKα-R may be attributable to incomplete stable hCKα knockdown, resulting in susceptibility to AL-9 inhibition. Therefore, hCKα upregulates PI4P production solely via PI4KIIIα activation during HCV replication. This notion supports the colocalization of hCKα with NS5A and PI4KIIIα or PI4P (Fig. 1).

Compared with the previous findings that NS5A interacts with PI4KIIIα and that NS5A and NS5B activate kinase activity at the viral replication site (24, 28), our results further emphasize that NS5A alone is insufficient to activate PI4KIIIα without the participation of hCKα. Therefore, hCKα may function as an indispensable coordinator to modulate NS5A-stimulated PI4KIIIα activation for massive PI4P production.

### hCKα activity upregulates PI4KIIIα activity in the absence of viral NS protein expression.

**FIG 4:**
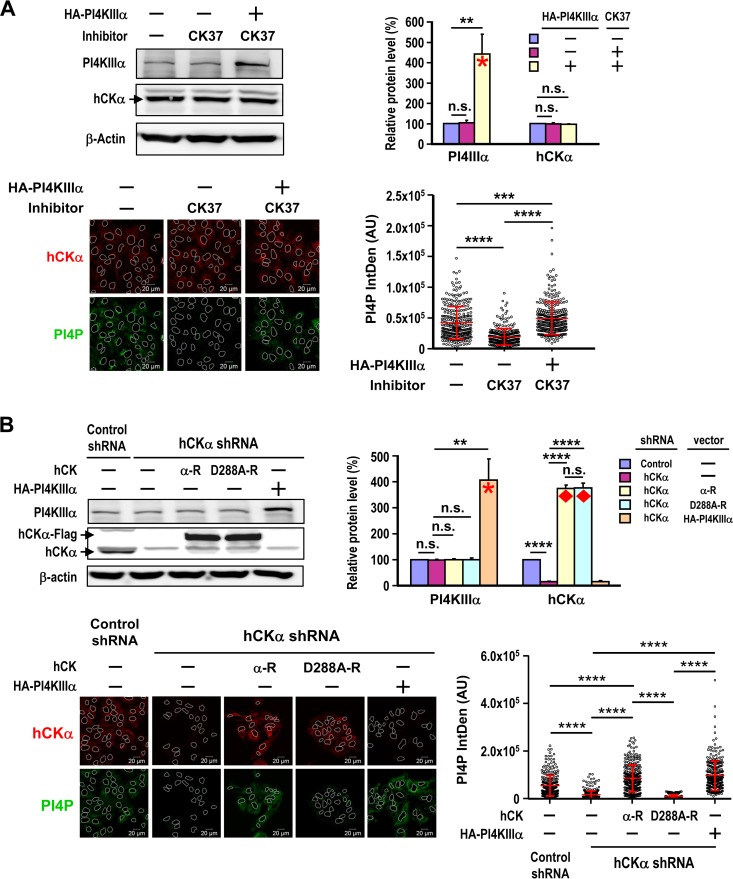
Effect of hCKα activity on PI4KIIIα activation without viral NS protein expression. (A) T7/Huh7 cells were transfected with the vector control plasmid or pTM-HA-PI4KIIIα and were then treated with a vehicle or CK37. (Top) The cells were then analyzed by Western blotting (left), and relative protein levels were quantified (right). The red asterisk indicates total levels of overexpressed and endogenous PI4KIIIα, which was detected with rabbit anti-PI4KIIIα by Western blotting, relative to the endogenous PI4KIIIα levels obtained in cells without CK37 treatment and HA-PI4KIIIα overexpression. **, *P* < 0.01; ***, *P* < 0.001; ****, *P* < 0.0001; n.s., nonsignificant. (Bottom) Another set of cells was monitored (left), and PI4P levels were quantitated (right). (B) Paired T7-control shRNA/Huh7 and T7-hCKα shRNA/Huh7 cells were transfected with either a control vector, a wild-type or D288A mutant hCKα-R plasmid, or pTM-HA-PI4KIIIα. The cells were then analyzed by Western blotting (top), and PI4P levels were quantitated (bottom). The red asterisk indicates the total levels of overexpressed and endogenous PI4KIIIα relative to endogenous PI4KIIIα levels obtained in control stable knockdown cells, whereas the red diamonds indicate the levels of overexpressed wild-type or D288A mutant hCKα-R relative to endogenous hCKα levels in control stable knockdown cells.

Because stable hCKα knockdown also decreased PI4P levels in cells without expression of viral proteins NS3 to NS5B, we next examined whether hCKα inactivation inhibits PI4P production and whether PI4KIIIα overexpression compensates for the defective PI4P production induced by CK37 in the absence of viral NS protein expression. HA-PI4KIIIα overexpression (Fig. 4A, top) ameliorated the inhibitory effects of CK37 on basal PI4P production (Fig. 4A, bottom), confirming that hCKα activity modulates basal PI4KIIIα activity, even in the absence of NS3 to NS5B expression.

Next, we performed a rescue analysis to confirm the direct effect of hCKα activity on PI4KIIIα activation. As a positive control, HA-PI4KIIIα overexpression (Fig. 4B, top) rescued PI4P accumulation in hCKα stable knockdown cells (Fig. 4B, bottom), again indicating that hCKα-induced PI4P production is mediated by PI4KIIIα. Similarly, overexpression of wild-type (but not D288A mutant) hCKα-R (Fig. 4B, top) restored PI4P in hCKα stable knockdown cells to levels even higher than those detected in control stable knockdown cells (Fig. 4B, bottom). These findings illustrate that hCKα activity directly modulates PI4KIIIα activity, even in the absence of viral NS protein expression.

### hCKα does not have a role in PI4KIIIα expression.

**FIG 5:**
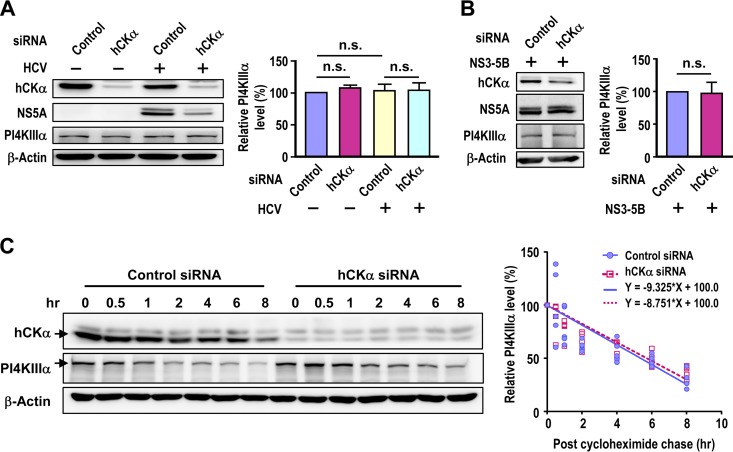
hCKα is not involved in PI4KIIIα expression. (A) Huh7 cells were transfected with control or hCKα siRNA and then either remained mock infected or were infected with HCVcc. (Left) The cells were then harvested and subjected to immunoblot detection. (Right) PI4KIIIα levels under different experimental conditions, relative to the level detected in control siRNA transfection without HCV infection, which was arbitrarily set at 100%, were quantitated. (B) T7/Huh7 cells were transfected first with untargeted or hCKα siRNA and then with pTM-NS3–5B. The cells were then subjected to Western blotting. n.s., nonsignificant. (C) Huh7 cells were transfected with control or hCKα siRNA and were then treated with cycloheximide. (Left) The cells were collected at different time points post-cycloheximide addition for Western blot analysis. (Right) PI4KIIIα levels were first normalized to those of β-actin. PI4KIIIα levels at different times, expressed as percentages relative to the level detected at time zero in each siRNA transfection, which was arbitrarily set to 100%, are shown. The data from 5 independent analyses were fit to a linear regression equation in order to assess the degradation rate of PI4KIIIα in control or hCKα transient knockdown cells.

Because hCKα plays a crucial role in elevated PI4P production, we next investigated whether the inactivation or depletion of hCKα affects PI4KIIIα expression and subsequently inhibits PI4P production. We showed above that CK37 and AL-9 did not affect PI4KIIIα levels in HCV-infected cells (Fig. 2C). hCKα knockdown by specific siRNAs did not exert obvious effects on PI4KIIIα levels in mock- and HCV-infected cells (Fig. 5A). Additionally, comparable amounts of PI4KIIIα were detected in NS3–5B-expressing cells transfected with untargeted or hCKα siRNAs (Fig. 5B). Furthermore, a cycloheximide chase experiment revealed indistinguishable degradation rates for PI4KIIIα in control and hCKα siRNA-transfected cells (Fig. 5C). These results collectively show that hCKα does not affect PI4KIIIα expression.

### PI4KIIIα inactivation impairs the colocalization of hCKα with PI4KIIIα and NS5A on the ER membrane.

We demonstrated previously that hCKα activity is required for the efficient translocation of hCKα and NS5A to the ER (35) and showed above that the inactivation of hCKα or PI4KIIIα by CK37 or AL-9 reduced HCV-mediated PI4P accumulation (Fig. 2D). To provide evidence for the functional link between hCKα and PI4KIIIα, we examined whether PI4KIIIα inactivation exhibited a phenotype similar to that achieved with hCKα knockdown, which impairs NS5A transport to the ER (35), and whether PI4KIIIα inactivation also hindered PI4KIIIα translocation to the ER, because PI4KIIIα and NS5A localization sites on the ER are indicative of viral replication factories (24, 42).

**FIG 6:**
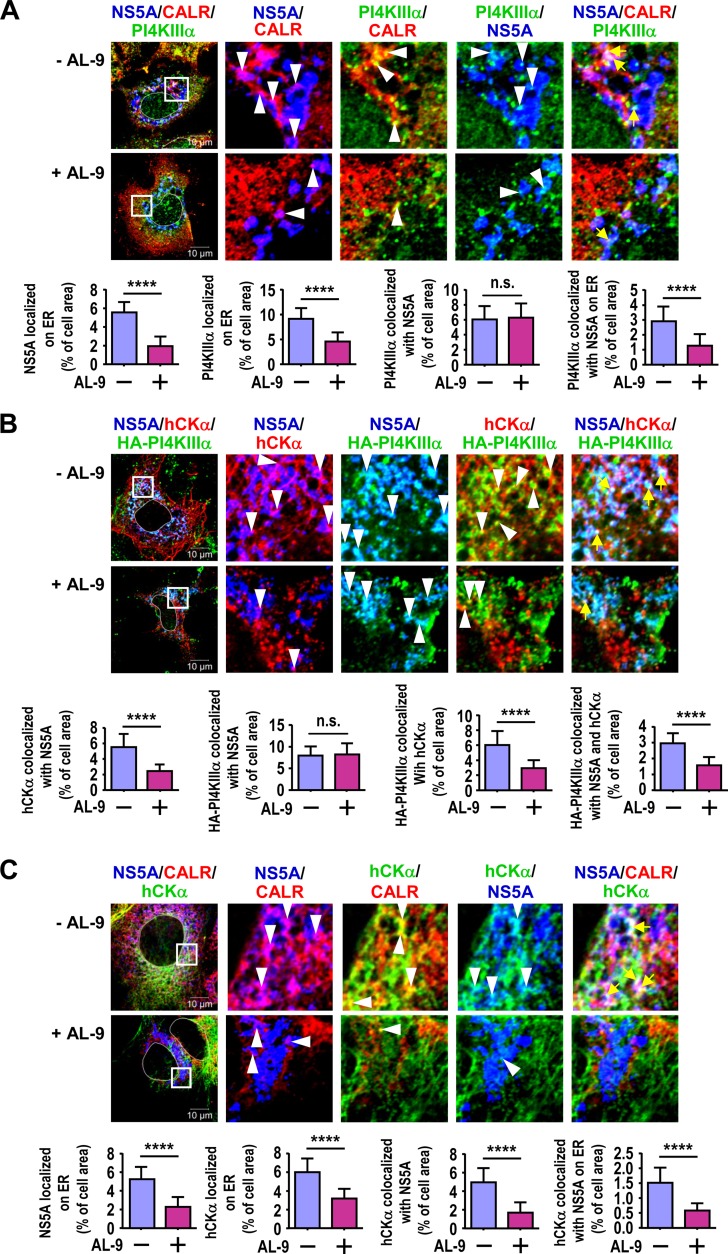
Effects of PI4KIIIα inactivation on the translocation of hCKα, NS5A, and PI4KIIIα to the ER. (A) T7/Huh7 cells were first transfected with pTM-NS3–5B and then treated with a vehicle or AL-9. The cells were fixed and stained with MAb 9E10, rabbit anti-PI4KIIIα, and goat anti-CALR, followed by incubation with the appropriate Alexa Fluor-conjugated secondary antibodies. The cells were then examined by confocal microscopy. (B) T7/Huh7 cells were cotransfected with pTM-HA-PI4KIIIα and pTM-NS3–5B and were then treated with a vehicle or AL-9. The cells were immunostained with MAb 9E10, rabbit anti-hCKα, and goat anti-HA and were examined by confocal microscopy. (C) T7/Huh7 cells were first transfected with pTM-NS3–5B and then treated with a vehicle or AL-9. The cells were immunostained with MAb 9E10, rabbit anti-hCKα, and goat anti-CALR and were then analyzed by confocal microscopy. In each analysis, a representative image of the fluorescence signals is shown. The boxed area shown in the merged image was enlarged, and the colocalization of the two or three indicated proteins is marked with white arrowheads or yellow arrows, respectively. The colocalization of the indicated proteins was quantitated by determining the colocalization area of the indicated molecules relative to the entire cell area, which is shown in each bar graph. ****, *P* < 0.0001; n.s., nonsignificant.

Treatment with AL-9 (or CK37 in hCKα inactivation) interferes with viral RNA replication and complicates the detection of NS5A on the ER; therefore, a transient pTM-NS3–5B expression model was employed. T7/Huh7 cells overexpressing NS3–5B were treated with a vehicle or AL-9, and the subcellular distribution of PI4KIIIα and NS5A and their localization on the ER, which was immunostained using anti-calreticulin (also known as calregulin [CALR], an ER-resident marker), were examined via confocal microscopy. Quantitative analysis of 20 randomly selected cells indicated that AL-9 treatment not only hindered the distribution of NS5A on the ER but also reduced the localization of PI4KIIIα on the ER from that achieved with vehicle treatment (Fig. 6A). In addition, AL-9 treatment hindered the ER colocalization of these two molecules (Fig. 6A). However, PI4KIIIα still effectively colocalized with NS5A regardless of AL-9 treatment (Fig. 6A).

We then investigated whether AL-9 affected the colocalization of hCKα with overexpressed HA-PI4KIIIα and NS5A. AL-9 decreased the colocalization of hCKα with NS5A or with HA-PI4KIIIα, as well as the colocalization of hCKα with both HA-PI4KIIIα and NS5A (Fig. 6B). Because PI4KIIIα activity was required for the efficient transport of PI4KIIIα and NS5A to the ER membrane (Fig. 6A) as well as for the colocalization of hCKα with HA-PI4KIIIα and NS5A (Fig. 6B), we sought to determine whether PI4KIIIα activity is also important for the colocalization of hCKα with NS5A on the ER. As shown in Fig. 6C, treatment with AL-9 not only impeded hCKα localization on the ER but also interfered with the colocalization of hCKα and NS5A, as well as with the colocalization of these proteins on the ER.

Collectively, these data show that in addition to the PI4KIIIα-regulated translocation of PI4KIIIα and NS5A to the ER, the trafficking of hCKα to the ER is regulated by PI4KIIIα activity. These results also indicate that PI4KIIIα activity is required for the effective colocalization of hCKα with PI4KIIIα and NS5A on the ER, whereas it is not crucial for PI4KIIIα and NS5A colocalization.

### Inactivation of hCKα interferes with the translocation of hCKα, PI4KIIIα, and NS5A to the ER membrane.

**FIG 7:**
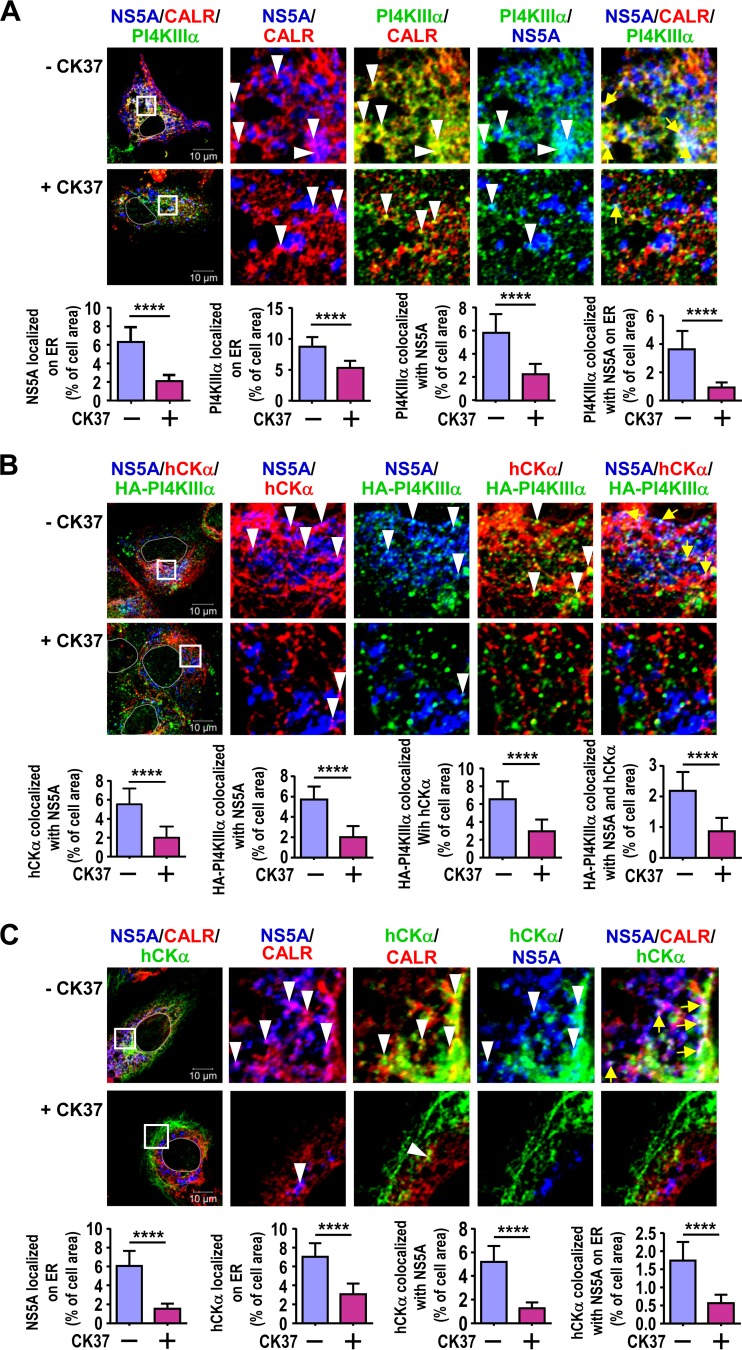
Effects of hCKα inactivation on the translocation of PI4KIIIα and NS5A to the ER. (A) T7/Huh7 cells were transfected with pTM-NS3–5B, followed by treatment with a vehicle or CK37. The cells were incubated with MAb 9E10, rabbit anti-PI4KIIIα, and goat anti-CALR and were then analyzed by confocal microscopy. (B) T7/Huh7 cells were cotransfected with pTM-HA-PI4KIIIα and pTM-NS3–5B, followed by vehicle or CK37 treatment. The cells were immunostained with MAb 9E10, rabbit anti-hCKα, and goat anti-HA and were then analyzed by confocal microscopy. (C). T7/Huh7 cells were first transfected with pTM-NS3–5B and then treated with a vehicle or CK37. The cells were immunostained with MAb 9E10, rabbit anti-hCKα, and goat anti-CALR prior to confocal microscopy. In each study, fluorescence images were analyzed, and colocalization of the indicated proteins was quantified as described in the legend to Fig. 6. ****, *P* < 0.0001.

To further address the functional relationship between hCKα and PI4KIIIα, we examined the effect of hCKα inactivation on the colocalization of PI4KIIIα and NS5A on the ER. In agreement with our previous finding that hCKα activity is crucial for the transport of NS5A to the ER (35), CK37 treatment hindered the localization of PI4KIIIα on the ER (Fig. 7A). Additionally, CK37 not only inhibited the colocalization of PI4KIIIα with NS5A but also interfered with the colocalization of these proteins on the ER (Fig. 7A). In addition to inhibiting the colocalization of hCKα with NS5A, CK37 treatment also interfered with the colocalization of HA-PI4KIIIα with hCKα and NS5A (Fig. 7B). Moreover, CK37 treatment impaired the colocalization of hCKα with NS5A on the ER (Fig. 7C). Therefore, the results shown in Fig. 7 collectively demonstrate that hCKα activity plays a critical role in the ER translocation of hCKα and NS5A and is also necessary for the effective translocation of PI4KIIIα to the ER.

### Inactivation of hCKα and PI4KIIIα does not affect the localization of NS5B on the ER.

**FIG 8:**
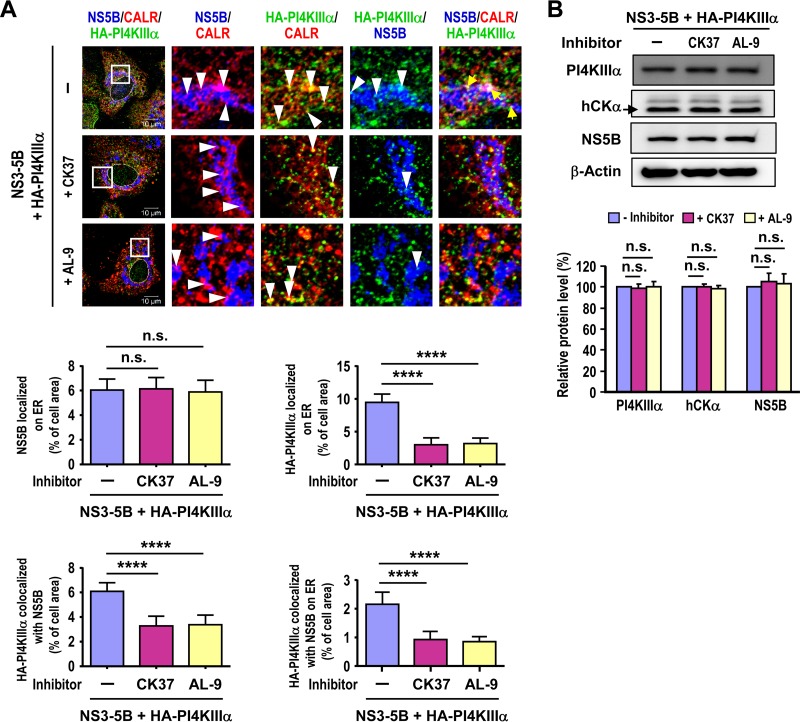
Examination of the effects of PI4KIIIα or hCKα inactivation on the localization of NS5B on the ER. (A) T7/Huh7 cells were cotransfected with pTM-HA-PI4KIIIα and pTM-NS3–5B, followed by treatment with a vehicle, AL9, or CK37. The cells were immunostained with mouse anti-NS5B, rabbit anti-HA, and goat anti-CALR and were then analyzed by confocal microscopy. Fluorescence images were analyzed to quantify the colocalization of the indicated proteins. ****, *P* < 0.0001; n.s., nonsignificant. (B) Another set of samples was analyzed by Western blotting (top), and the relative protein levels were quantified (bottom).

We demonstrated previously that the ER localization of NS5A (but not that of NS5B or NS3) is inhibited by hCKα knockdown (35). We next investigated whether the inhibitory effects of hCKα or PI4KIIIα inactivation on the translocation of NS5A to the ER are specific by evaluating the effects of hCKα or PI4KIIIα inactivation on the ER localization of NS5B. In contrast to the inhibitory effects of AL-9 and CK37 on the localization of HA-PI4KIIIα on the ER, AL-9 or CK37 did not affect the localization of NS5B on the ER (Fig. 8A). However, the colocalization of NS5B with HA-PI4KIIIα and the colocalization of these proteins on the ER were inhibited by AL-9 or CK37 (Fig. 8A). As an internal control, Western blot analysis indicated that CK37 or AL-9 treatment did not affect levels of PI4KIIIα, hCKα, or NS5B (Fig. 8B). Based on these observations, we conclude that NS5B localizes on the ER even when PI4KIIIα is redirected to non-ER membranes due to the disruption of PI4P synthesis.

### hCKα forms a ternary complex with PI4KIIIα and NS5A.

**FIG 9:**
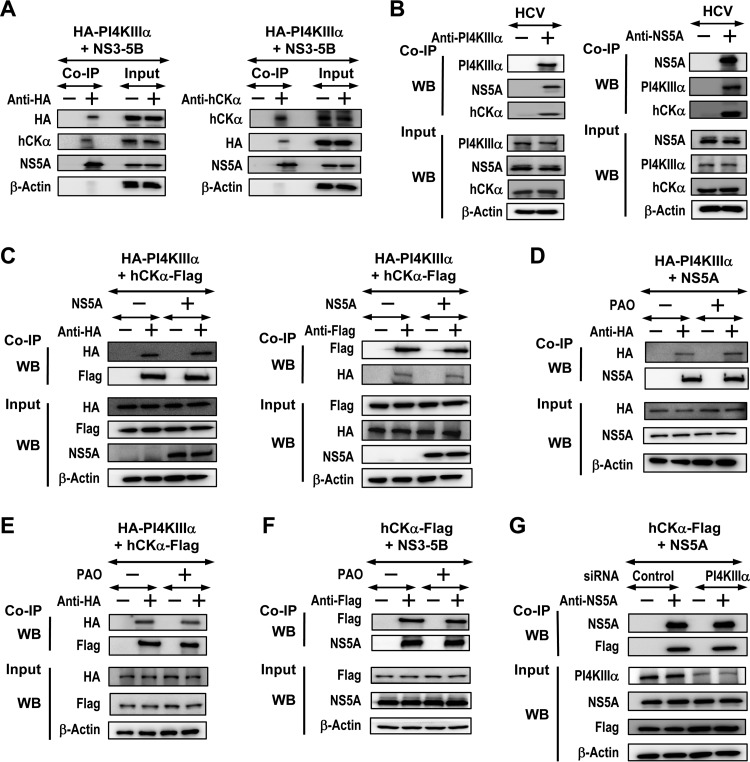
Analysis of the formation of the hCKα–NS5A–PI4KIIIα ternary complex and the effects of NS5A, PAO, and PI4KIIIα knockdown on protein-protein interactions. (A) T7/Huh7 cells were cotransfected with pTM-HA-PI4KIIIα and pTM-NS3–5B. The cell lysates were incubated with rabbit isotype-matched control IgG (−) or rabbit anti-HA (left) or with rabbit isotype control IgG or rabbit anti-hCKα (right). The precipitated proteins were subjected to immunoblot analysis. A portion of the cell lysates corresponding to 5% of the total proteins used for co-IP was also loaded as an input control in the Western blot analysis. (B) Lysates obtained from HCV-infected cells were incubated with rabbit control isotype IgG or anti-PI4KIIIα (left) or with mouse control isotype IgG or anti-NS5A (right), and the precipitated proteins were subjected to Western blot detection. (C) T7/Huh7 cells were cotransfected with pTM-HA-PI4KIIIα and pCMV6-hCKα with or without pTM-NS5A. The cell lysates were incubated with rabbit anti-HA (left) or rabbit anti-Flag (right). The isolated proteins were subjected to immunoblot analysis. (D and E) T7/Huh7 cells were cotransfected with pTM-HA-PI4KIIIα and pTM-NS5A (D) or with pTM-HA-PI4KIIIα and pCMV6-hCKα (E). The cells were treated with a vehicle or PAO, and the cell lysates were incubated with rabbit anti-HA prior to Western blot analysis. (F) T7/Huh7 cells were cotransfected with pTM-NS3–5B and pCMV6-hCKα, followed by vehicle or PAO treatment. Cell lysates were precipitated with rabbit anti-Flag prior to immunoblot analysis. (G) T7/Huh7 cells were first transfected with nontargeted siRNA or PI4KIIIα siRNA, followed by transfection with pCMV6-hCKα and pTM-NS5A. The lysates from transfected cells were immunoprecipitated with mouse anti-NS5A prior to Western blot analysis. In panels C to G, the results from three independent analyses did not demonstrate statistically significant differences between the vehicle and PAO treatment or between control and tested conditions. Therefore, representative sets of data are shown.

In order to understand the molecular functions underlying the role of hCKα in the production of PI4P and the ER trafficking of hCKα, PI4KIIIα, and NS5A, we performed coimmunoprecipitation (co-IP) to examine whether these three functionally interrelated proteins form a complex in cells. Lysates from cells overexpressing NS3–5B and HA-PI4KIIIα were incubated with a rabbit isotype IgG or anti-HA, and the precipitated proteins were subjected to immunoblot analysis. HA-PI4KIIIα was specifically precipitated by anti-HA but not by control IgG (Fig. 9A, left). Anti-HA also specifically pulled down hCKα as well as NS5A (Fig. 9A, left). Additionally, the precipitation of hCKα with rabbit anti-hCKα specifically cocaptured HA-PI4KIIIα as well as NS5A (Fig. 9A, right).

To confirm ternary complex formation in HCV-infected cells, lysates from HCV-infected cells were incubated with rabbit isotype IgG or anti-PI4KIIIα, followed by Western blotting. Precipitation of PI4KIIIα with anti-PI4KIIIα also specifically pulled down NS5A and hCKα (Fig. 9B, left). Additionally, anti-NS5A not only precipitated NS5A but also cocaptured PI4KIIIα and hCKα (Fig. 9B, right). Thus, hCKα, PI4KIIIα, and NS5A are interacting partners that form the ternary complex.

### hCKα binds PI4KIIIα independently of NS5A.

Because hCKα was found to colocalize with PI4KIIIα in the absence of NS5A, and because NS5A expression augmented the colocalization of hCKα and PI4KIIIα (Fig. 1C), we examined whether hCKα formed a complex with PI4KIIIα in the absence of NS5A and whether NS5A expression enhanced the binding of hCKα and PI4KIIIα. The precipitation of HA-PI4KIIIα with anti-HA from cells coexpressing HA-PI4KIIIα and hCKα tagged with a Flag epitope at its C terminus, i.e., hCKα-Flag, with or without NS5A coexpression, pulled down similar amounts of hCKα-Flag (Fig. 9C, left). Similarly, the precipitation of hCKα-Flag with anti-Flag cocaptured comparable amounts of HA-PI4KIIIα in the presence or absence of NS5A coexpression (Fig. 9C, right). These results indicate that NS5A does not have a crucial role in the hCKα-PI4KIIIα interaction.

### PI4KIIIα activity is not necessary for the binding of PI4KIIIα to NS5A or hCKα and is not critical for hCKα-Flag and NS5A interactions.

In order to determine the molecular events underlying the effects of PI4KIIIα inactivation on the transport of hCKα, PI4KIIIα, and NS5A to the ER (Fig. 6), phenylarsine oxide (PAO), an inhibitor of PI4KIIIα (28), was applied to investigate the binding of PI4KIIIα to NS5A or hCKα. PAO treatment did not affect the binding of HA-PI4KIIIα to NS5A (Fig. 9D) or hCKα-Flag (Fig. 9E). In addition, PAO did not affect the binding of hCKα-Flag to NS5A (Fig. 9F), a finding consistent with the observation that PI4KIIIα knockdown did not exert any apparent effects on the hCKα-Flag and NS5A interaction (Fig. 9G). These results collectively indicate that PI4KIIIα activity is not involved in the interactions between any two components of the ternary complex.

### Although hCKα does not have an apparent effect on the interaction of hCKα with PI4KIIIα or NS5A, its activity facilitates the binding of PI4KIIIα to NS5A.

**FIG 10:**
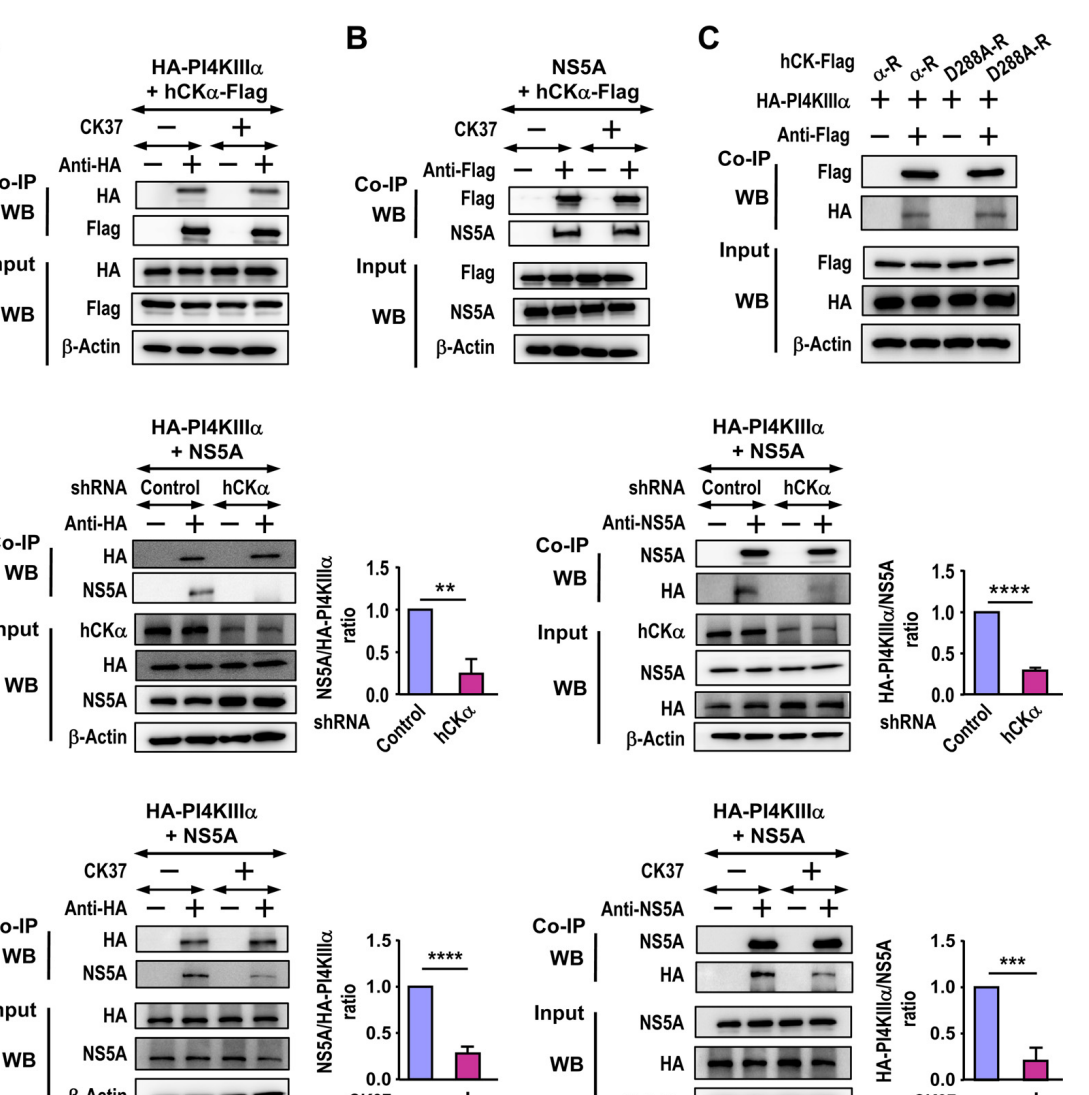
Effects of CK37 and hCKα knockdown on protein-protein interactions. (A and B) T7/Huh7 cells were cotransfected with pTM-HA-PI4KIIIα and pCMV6-hCKα (A) or with pTM-NS5A and pCMV6-hCKα (B). The cells were then treated with a vehicle or CK37, and cell lysates were incubated with rabbit isotype IgG or anti-HA (A) or with rabbit isotype IgG or anti-Flag (B). The precipitated proteins were then analyzed by Western blotting. (C) T7/Huh7 cells were cotransfected with pTM-HA-PI4KIIIα and wild-type or D288A mutant hCKα-R. The cell lysates were then incubated with rabbit anti-Flag, and the precipitated proteins were subjected to Western blot analysis. (D) T7-control shRNA/Huh7 and T7-hCKα shRNA/Huh7 cells were cotransfected with pTM-HA-PI4KIIIα and pTM-NS5A. The cell lysates were incubated with rabbit anti-HA (left) or an anti-NS5A MAb (right) prior to Western blot analysis. The binding of the two coimmunoprecipitated proteins, as indicated in the quantitation graphs, obtained from hCKα stable knockdown cells was expressed as a ratio relative to the binding obtained from control stable knockdown cells, which was arbitrarily set at a ratio of 1. (E) T7/Huh7 cells were cotransfected with pTM-HA-PI4KIIIα and pTM-NS5A, followed by treatment with a vehicle or CK37 as indicated. The cell lysates were then incubated with rabbit anti-HA (left) or an anti-NS5A MAb (right), and the precipitated proteins were analyzed by immunoblot detection. The binding of the two coprecipitated proteins, as indicated in each quantitation graph, obtained from vehicle-treated cells was arbitrarily assigned a ratio of 1, and binding obtained from CK37 treatment was expressed as a relative ratio. In panels A to C, the results from three separate studies did not demonstrate statistically significant differences between vehicle and CK37 treatment or between the wild type and D288A mutant hCKα-R expression. Therefore, representative sets of data are shown. **, *P* < 0.01; ***, *P* < 0.001; ****, *P* < 0.0001.

To understand the molecular basis of the effects of hCKα activity on the ER translocation of hCKα, PI4KIIIα, and NS5A (Fig. 7), we examined whether CK37 affected the binding of hCKα to PI4KIIIα or NS5A. According to co-IP analyses, CK37 did not affect the binding of hCKα-Flag to HA-PI4KIIIα (Fig. 10A) or alter the hCKα-Flag and NS5A interaction (Fig. 10B). Additionally, wild-type hCKα-R and the D288A mutant bound comparably to HA-PI4KIIIα (Fig. 10C), supporting the former observation.

Next, we examined whether hCKα is involved in the PI4KIIIα-NS5A interaction. The precipitation of HA-PI4KIIIα from lysates containing HA-PI4KIIIα and NS5A with anti-HA pulled down a barely detectable NS5A band in hCKα stable knockdown cells, in contrast to the band obtained from control stable knockdown cells (Fig. 10D, left). Conversely, the precipitation of NS5A with anti-NS5A from hCKα stable knockdown cells cocaptured a barely visible HA-PI4KIIIα band, in contrast to the band observed in control stable knockdown cells (Fig. 10D, right). Quantitation of the data from three independent studies indicated that hCKα stable knockdown reduced the ratios of NS5A binding to HA-PI4KIIIα and HA-PI4KIIIα binding to NS5A to approximately 0.24 and 0.28, respectively, relative to that for control stable knockdown cells, which was arbitrarily designated 1 (Fig. 10D).

Because CK37 reduces the colocalization of PI4KIIIα and NS5A (Fig. 7A), we then determined whether the binding of PI4KIIIα to NS5A requires hCKα activity. When cell lysates containing HA-PI4KIIIα and NS5A were incubated with anti-HA or anti-NS5A, smaller amounts of NS5A or HA-PI4KIIIα coprecipitated with HA-PI4KIIIα or NS5A, respectively, in CK37-treated cells than in vehicle-treated cells (Fig. 10E, left and right, respectively). Quantitation of the data from three separate analyses indicated that CK37 treatment reduced the ratios of NS5A binding to HA-PI4KIIIα and HA-PI4KIIIα binding to NS5A to approximately 0.28 and 0.21 relative to that for vehicle treatment, which was arbitrarily designated 1 (Fig. 10E, left and right, respectively). These results indicate that hCKα activity promotes the recruitment of PI4KIIIα to NS5A.

### hCKα upregulates PI4KIIIα activity *in vitro* in the presence or absence of NS5A.

**FIG 11:**
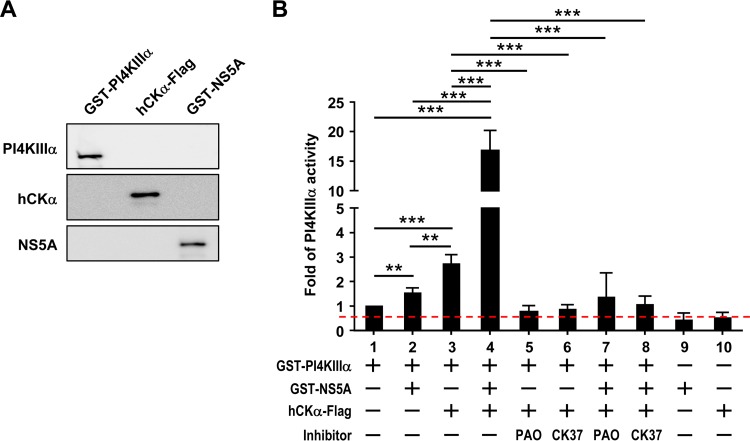
Upregulation of PI4KIIIα activity *in vitro* by hCKα. (A) Thirty nanograms each of recombinant PI4KIIIα, hCKα, and NS5A proteins was analyzed by Western blotting using rabbit anti-PI4KIIIα, rabbit anti-hCKα, and an anti-NS5A MAb, respectively. (B) The *in vitro* activity of purified recombinant PI4KIIIα was assayed in the presence or absence of recombinant NS5A, hCKα, and/or inhibitors in an ADP-Glo kinase assay as indicated. Kinase activity, monitored as the conversion of ATP to ADP under different experimental conditions relative to that detected in PI4KIIIα alone, which was arbitrarily set at a value of 1, was expressed as the fold change in luciferase activity. The dashed red line indicates the threshold for this *in vitro* kinase assay. The data shown are the quantitated results from four independent experiments. **, *P* < 0.01; ***, *P* < 0.001.

To further support the role of hCKα in PI4KIIIα activation, the *in vitro* activity of recombinant PI4KIIIα was assayed in the presence or absence of recombinant NS5A and/or hCKα. Recombinant PI4KIIIα, obtained from the baculovirus expression system, comprises the amino acid residues between positions 1249 and 2102 in the kinase, including the kinase catalytic domain. Recombinant NS5A, purified from Escherichia coli, comprises amino acids 2061 to 2302 in the HCV polyprotein, i.e., the C-terminal portion of domain 1, low-complexity sequence 1, and the N-terminal portion of domain 2 of NS5A. Both recombinant proteins are fused with glutathione *S*-transferase (GST) at their N termini, and their expected molecular masses are 124 kDa (for PI4KIIIα) and 53 kDa (for NS5A). Recombinant hCKα was purified from 293T cells transfected with pCMV6-hCKα. The authenticity of these proteins was confirmed by immunoblotting (Fig. 11A). As expected, the addition of NS5A to PI4KIIIα increased PI4KIIIα activity (Fig. 11B, compare bars 1 and 2). The addition of hCKα to PI4KIIIα increased PI4KIIIα basal activity to levels even higher than those activated by NS5A (Fig. 11B, compare bar 1 with bars 2 and 3). Remarkably, hCKα, in conjunction with NS5A, greatly augmented PI4KIIIα activation (Fig. 11B, compare bars 2 and 4). hCKα-stimulated basal PI4KIIIα activity was inhibited by both PAO (Fig. 11B, compare bars 3 and 5) and CK37 (Fig. 11B, compare bars 3 and 6). Likewise, hCKα-augmented PI4KIIIα activity triggered by NS5A was ablated by PAO (Fig. 11B, compare bars 4 and 7) and CK37 (Fig. 11B, compare bars 4 and 8). Based on these results, we conclude that hCKα activity directly stimulates *in vitro* PI4KIIIα activity and that ternary complex formation is essential for robust PI4KIIIα activation.

### PI4KIIIα overexpression rescues the impaired viral replication and PI4P production caused by hCKα inactivation.

**FIG 12:**
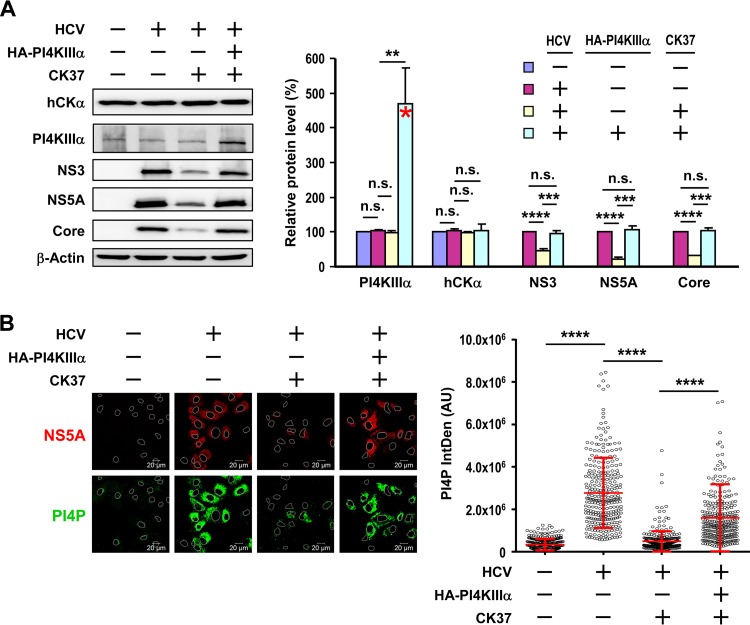
Rescue of viral replication and PI4P production by PI4KIIIα overexpression in CK37-treated, HCV-infected cells. (A) T7/Huh7 cells remained uninfected or were infected with HCV; they were then transfected with the control vector or pTM-HA-PI4KIIIα and were treated with the vehicle or CK37. (Left) The cells were then analyzed by Western blotting. (Right) PI4KIIIα and hCKα levels under the tested conditions relative to those detected in mock-infected cells without HA-PI4KIIIα overexpression or CK37 treatment were determined. The red asterisk marks the total levels of overexpressed and endogenous PI4KIIIα, which were detected using anti-PI4KIIIα in a Western blot, relative to endogenous PI4KIIIα levels in mock-infected cells without overexpression or CK37 treatment, expressed as a percentage. The levels of NS3, NS5A, and core under different conditions, relative to the levels detected in HCV-infected cells without overexpression or CK37 treatment, are also shown. (B) Another set of cells, as described for panel A, was monitored for the intracellular localization of NS5A and PI4P (left), and PI4P levels were quantitated (right). **, *P* < 0.01; ***, *P* < 0.001; ****, *P* < 0.0001; n.s., nonsignificant.

Because hCKα effectively interacted with PI4KIIIα regardless of PI4KIIIα or hCKα inactivation (Fig. 9E and 10A and C), we next examined the functional interaction between these two proteins and tested whether PI4KIIIα overexpression counteracts the negative effects of CK37 on viral replication in HCV-infected cells. Total levels of overexpressed HA-PI4KIIIα and endogenous PI4KIIIα were approximately 4.7-fold greater than the endogenous PI4KIIIα levels detected in mock-infected cells without CK37 treatment or HA-PI4KIIIα overexpression (Fig. 12A). The overexpression of PI4KIIIα in CK37-treated, HCV-infected cells resulted in levels of viral proteins, such as NS3, NS5A, and Core, higher than those in CK37-treated, HCV-infected cells without PI4KIIIα overexpression (Fig. 12A). Additionally, the levels of these viral proteins were comparable to those detected in HCV-infected cells treated with a vehicle (Fig. 12A). Based on these results, we conclude that PI4KIIIα overexpression compensates for the defects associated with hCKα inactivation during viral replication.

To determine whether hCKα modulates viral replication by activating PI4KIIIα for mass PI4P production, we determined whether PI4KIIIα overexpression could rescue PI4P production in CK37-treated HCV-infected cells. In agreement with the restoration of viral replication, the overexpression of PI4KIIIα substantially augmented PI4P production in HCV-infected cells treated with CK37 (Fig. 12B). These findings, taken together, indicate that the deficiency in hCKα activity observed during suppressed viral replication proceeds via attenuation of PI4KIIIα-mediated PI4P production.

### Expression of active (but not inactive) hCKα promotes the translocation of PI4KIIIα and NS5A to the ER in hCKα stable knockdown cells.

**FIG 13:**
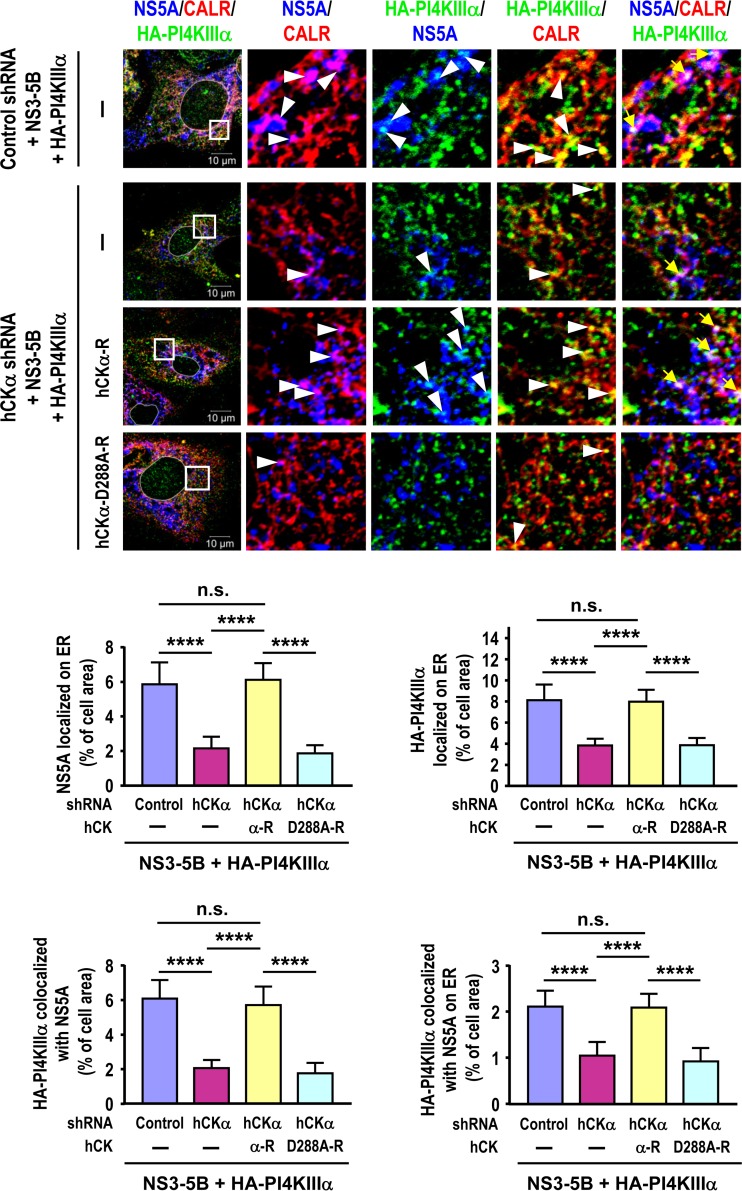
Effects of overexpression of the wild-type and D288A mutant hCKα-R proteins on the restoration of the ER translocation of PI4KIIIα and NS5A in hCKα stable knockdown cells. T7-control shRNA/Huh7 and T7-hCKα shRNA/Huh7 cells were cotransfected with pTM-NS3–5B and pTM-HA-PI4KIIIα along with the vector plasmid, wild-type hCKα-R, or D288A mutant hCKα-R. The cells were processed for confocal microscopy using MAb 9E10, goat anti-CALR, and rabbit anti-HA. (Top) Set of representative fluorescence images. (Bottom) Quantitation of the colocalization of the proteins. ****, *P* < 0.0001; n.s., nonsignificant.

According to our results, hCKα inactivation interferes with the translocation of PI4KIIIα and NS5A to the ER (Fig. 7); therefore, we next investigated whether hCKα activity directly upregulates the ER translocation of PI4KIIIα and NS5A in hCKα stable knockdown cells. In agreement with the inhibitory effects of CK37 on the ER translocation of NS5A and PI4KIIIα, stable hCKα knockdown also reduced the localization of NS5A and HA-PI4KIIIα on the ER (Fig. 13). Overexpression of wild-type (but not D288A mutant) hCKα-R in hCKα stable knockdown cells not only restored NS5A localization on the ER, in agreement with our previous observations (35), but also rescued the translocation of HA-PI4KIIIα to the ER (Fig. 13). Moreover, overexpression of wild-type (but not D288A mutant) hCKα-R restored the colocalization of NS5A and HA-PI4KIIIα on the ER (Fig. 13). These results, together with our previous findings (35), demonstrate that hCKα activity positively regulates the translocation of hCKα as well as NS5A and PI4KIIIα to the ER.

### Overexpression of active (but not inactive) PI4KIIIα restores the translocation of PI4KIIIα and NS5A to the ER in CK37-treated cells.

**FIG 14:**
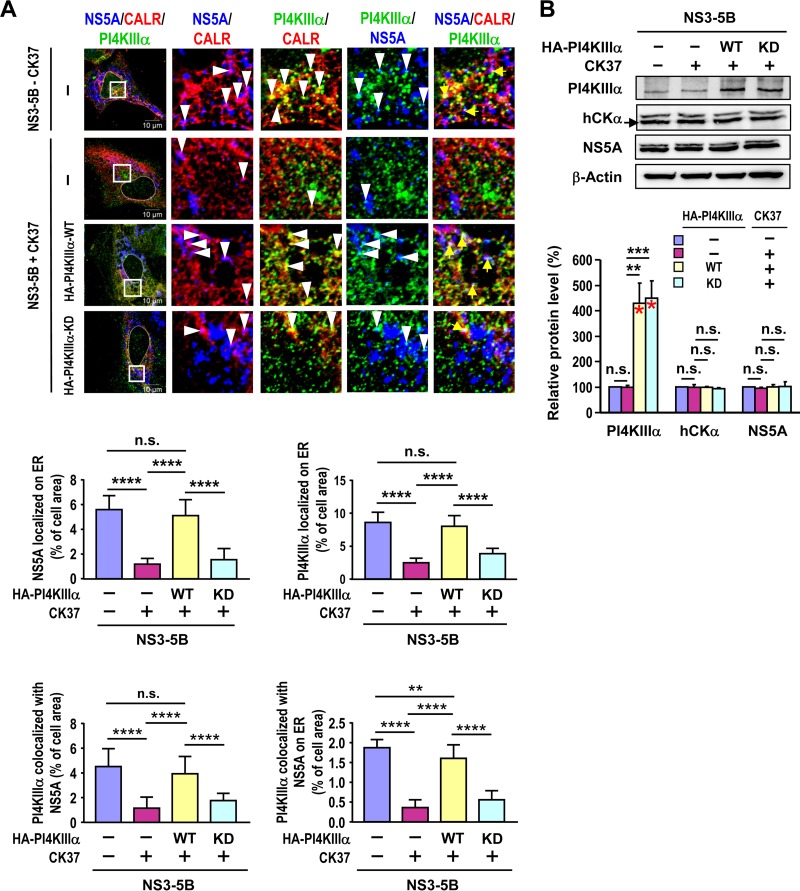
Effects of the overexpression of active and kinase-deficient PI4KIIIα proteins on the rescue of impaired ER translocation of PI4KIIIα and NS5A induced by CK37. (A) T7/Huh7 cells were cotransfected with pTM-NS3–5B and the control plasmid, pEF1A-HA-PI4KIIIα-WT, or pEF1A-HA-PI4KIIIα-KD, followed by vehicle or CK37 treatment. For the positive control for the ER translocation of PI4KIIIα and NS5A, a set of cells cotransfected with pTM-NS3–5B and the control vector was treated with the vehicle. The cells were processed for confocal microscopy using MAb 9E10, goat anti-CAL, and rabbit anti-PI4KIIIα (for cells without HA-PI4KIIIα overexpression) or rabbit anti-HA (for HA-PI4KIIIα overexpression) to quantify the degree of translocation of PI4KIIIα and NS5A to the ER. (B) Another set of samples was analyzed by Western blotting (top), and the relative protein levels were quantified (bottom). The red asterisk marks the levels of overexpressed wild-type or KD mutant HA-PI4KIIIα plus endogenous PI4KIIIα relative to those observed in cells without overexpression or CK37 treatment. **, *P* < 0.01; ***, *P* < 0.001; ****, *P* < 0.0001; n.s., nonsignificant.

Treatment with AL-9 or CK37 attenuated the production of PI4P (Fig. 2D) and the translocation of PI4KIIIα and NS5A as well as hCKα to the ER (Fig. 6 and 7); therefore, we investigated whether PI4P production is essential for the ER translocation of PI4KIIIα and NS5A. To address this issue, we investigated the abilities of active and kinase-deficient (KD) (K1792L mutant) HA-PI4KIIIα to ameliorate the impaired ER translocation of PI4KIIIα and NS5A caused by CK37 in NS3–5B-expressing cells. The PI4KIIIα mutant is unable to produce PI4P or to support viral replication (28). In agreement with the results shown in Fig. 7, CK37 reduced the translocation of PI4KIIIα and NS5A to the ER (Fig. 14A), and overexpression of wild-type HA-PI4KIIIα, but not its kinase-deficient mutant (Fig. 14B), restored the translocation of PI4KIIIα and NS5A to the ER in CK37-treated cells (Fig. 14A). These results strongly indicate that PI4KIIIα-mediated PI4P production is a prerequisite for the translocation of PI4KIIIα and NS5A to the ER (also, see below).

## DISCUSSION

We previously demonstrated the critical function of hCKα in promoting HCV membranous RC formation and viral replication (35). In our present study, we further elucidate the mechanism underlying the regulatory roles of hCKα in HCV replication. Like PI4KIIIα, hCKα played a crucial role in PI4P production and viral replication, as shown by siRNA knockdown and inhibitor analyses. Additionally, both hCKα and PI4KIIIα activities were critical for the translocation of hCKα, PI4KIIIα, and NS5A to the ER-derived membrane, indicating that these two kinases and NS5A function in concert to facilitate their own translocation to the ER. Furthermore, hCKα activity was essential for the generation of elevated PI4P pools in HCV-expressing cells. The overexpression of wild-type (but not D288A mutant) hCKα-R restored PI4P production in hCKα stable knockdown cells, which is consistent with our previous finding that the active (but not the D288A mutant) hCKα-R can rescue viral replication (35). Moreover, wild-type hCKα-R-mediated PI4P accumulation was abrogated by AL-9, a PI4KIIIα-specific inhibitor. These results collectively indicate that the profound PI4P synthesis mediated by hCKα occurs primarily via the upregulation of NS5A-stimulated PI4KIIIα activation and that PI4P functions as a critical determinant for the effective trafficking of these three interacting partners (see below) to the ER membrane for productive viral replication.

hCKα is present predominantly as a cytosolic protein, and HCV infection or the expression of NS proteins facilitates the localization of hCKα to the ER (35). Given the multiple intracellular localization sites of NS5A in perinuclear membranes, such as those of the ER, Golgi complex, and lipid droplets (5, 43), as well as the localization sites of PI4KIIIα in the ER (9, 10), Golgi complex-associated membrane vesicles (13), and the nucleolus (14), it would be difficult to determine unambiguously where PI4KIIIα and NS5A localize when PI4P synthesis is interrupted by CK37 or AL-9. Here we used an ER-residing marker, CALR, to investigate the transport or “translocation” of PI4KIIIα, NS5A, and hCKα to the PI4P-enriched, ER-associated membrane, because HCV replication increases their localization to the CALR-associated membrane. In contrast, AL-9 or CK37 hinders their colocalization with CALR, indicating that interference with PI4P production detours their ER translation to other membranes, which are collectively referred to as “non-ER membranes,” because they are not associated with CALR. Although HCV replication induces the ER transport of only a portion of these three replication components, this process is biologically and virologically relevant, because their “ER translocation” correlates well with elevated PI4P production and viral replication.

In agreement with our previous observation that the ER localization of NS5B is not affected by hCKα knockdown (35), NS5B localization on the ER, as shown by its colocalization with the ER marker CALR, was not influenced by AL-9 or CK37. However, the colocalization of NS5B and PI4KIIIα and their colocalization on the ER were inhibited by AL-9 or CK37. Based on these observations, we conclude that NS5B still localizes on the ER membrane when PI4P synthesis is impaired by inhibitors, while PI4KIIIα localizes to non-ER membranes. Therefore, the transport of NS5A and NS5B to the ER is differentially regulated; the route that delivers NS5A to the ER is strictly dependent on PI4P production, whereas the localization of NS5B on the ER is independent of PI4P. Therefore, HCV replication specifically “redirects” PI4KIIIα, NS5A, and hCKα to the ER.

**FIG 15:**
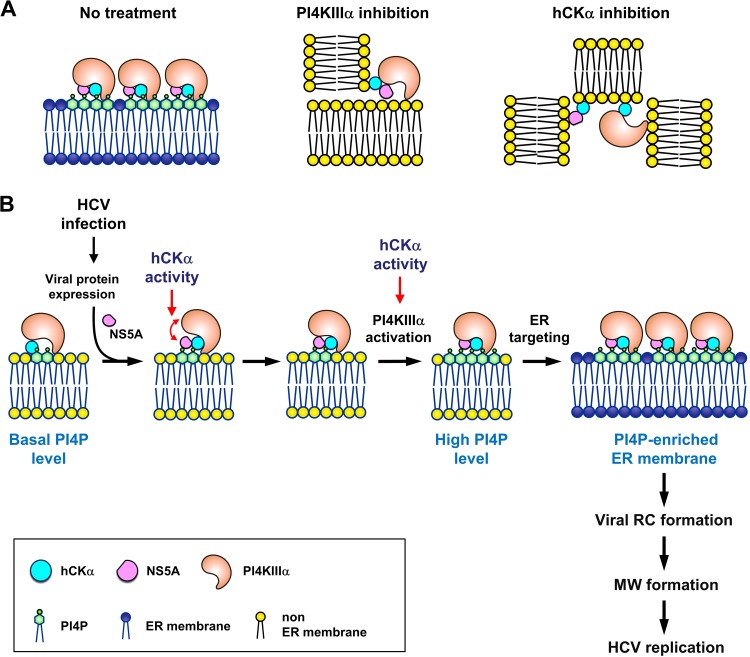
(A) Diagram showing the effects of AL-9 and CK37 on the colocalization of hCKα, PI4KIIIα, and NS5A on the ER membrane and the interactions of these three molecules. (Left) Formation of the ternary complex of hCKα, PI4KIIIα, and NS5A on the PI4P-enriched membrane derived from the ER in vehicle-treated cells. (Center) Effect of AL-9 on the segregation of hCKα from the PI4KIIIα-NS5A complex into two distinct non-ER membrane compartments. Nevertheless, the interaction between any of the two entities in the ternary complex is still intact. (Right) Effect of CK37 on the segregation of hCKα, PI4KIIIα, and NS5A into three distinct non-ER membranes. In this case, hCKα still binds PI4KIIIα and NS5A; however, PI4KIIIα and NS5A localize to different non-ER membranes, and their interaction is also disrupted. (B) Model illustrating the regulatory roles of hCKα in NS5A-stimulated PI4P production and the subsequent ER translocation of the hCKα, PI4KIIIα, and NS5A ternary complex. hCKα first interacts with PI4KIIIα at a non-ER membrane, where hCKα recruits and bridges NS5A and PI4KIIIα via hCKα activity, thus forming a ternary complex. The activity of PI4KIIIα in the ternary complex is synergistically upregulated by hCKα and NS5A, resulting in pronounced PI4P production. Additionally, hCKα activity directly upregulates PI4KIIIα activation without the participation of NS5A. These events then facilitate the targeting of hCKα, PI4KIIIα, and NS5A to the ER-derived membrane, where hCKα mediates the binding of NS5A to NS5B and, along with NS5A, activates PI4KIIIα, resulting in the formation of the PI4P-enriched platform for the continued viral replication complex (RC) assembly and membranous web (MW) formation necessary for viral replication.

Furthermore, we performed a series of co-IP analyses to elucidate the molecular basis of the function of hCKα in NS5A-stimulated PI4KIIIα activation and the translocation of these three molecules to the ER. The inactivation of PI4KIIIα by PAO or of hCKα by CK37 did not have an apparent effect on PI4KIIIα-hCKα binding. In support of this finding, the wild-type and D288A mutant hCKα-R clones interacted comparably with PI4KIIIα. Thus, the inability of the D288A mutant to rescue PI4P generation in hCKα stable knockdown cells expressing NS3–5B proteins is attributable to its lack of hCKα activity rather than to its inability to bind PI4KIIIα. In the present study, as well as in previous studies (35), we observed that D288A mutant hCKα still possesses wild-type-like NS5A-binding ability (35), and PAO or CK37 did not affect the interaction between these two molecules. In addition, hCKα interacted effectively with NS5A, even when PI4KIIIα was knocked down. Collectively, these results imply that PI4P levels do not influence hCKα binding to PI4KIIIα or NS5A. Nevertheless, AL-9 or CK37 reduced the colocalization of hCKα with NS5A or PI4KIIIα on the ER membrane. Thus, in the absence of massive PI4P production, the physical interactions between hCKα and PI4KIIIα and between hCKα and NS5A do not ensure the correct translocation of hCKα with PI4KIIIα or NS5A to the same ER membrane; instead, these paired molecules are spatially segregated into different non-ER membrane compartments (Fig. 15A, center and right).

Although AL-9 did not affect PI4KIIIα and NS5A colocalization, it inhibited the colocalization of these two molecules on the ER. This observation is consistent with the finding that the PI4KIIIα-NS5A interaction was not influenced by PAO, as shown in the present study, or by AL-9, as reported previously (29). Thus, in the absence of PI4KIIIα activity, PI4KIIIα and NS5A still colocalize and interact with each other on the same non-ER membrane (Fig. 15A, center). In contrast, CK37 inhibited the colocalization of these two proteins, a finding compatible with the co-IP results showing that hCKα knockdown or inactivation by CK37 impaired PI4KIIIα-NS5A binding. In the absence of hCKα activity, hCKα, PI4KIIIα, and NS5A appear to segregate into different non-ER membrane compartments in which hCKα still binds to PI4KIIIα and NS5A (Fig. 15A, right). Conversely, the paradoxical discrepancy between the differential effects of NS5A on the colocalization of PI4KIIIα with hCKα and the binding of PI4KIIIα to hCKα may be interpreted as follows. In the absence of NS5A, hCKα-PI4KIIIα complexes do not accumulate on membranes devoid of PI4P, thus increasing the difficulty in visualizing these complexes via microscopy. However, NS5A expression enhances the translocation of the hCKα-PI4KIIIα-NS5A complex to the ER, resulting in the formation and accumulation of viral RCs on the PI4P-enriched membrane (Fig. 15B) and increasing the visibility of these complexes by confocal microscopy over that of the single hCKα-PI4KIIIα complex.

The differential effects of CK37 and PAO on the colocalization and interaction of PI4KIIIα and NS5A are not attributable to the influence of PI4P production on the PI4KIIIα-NS5A interaction. Instead, they may be ascribed to the specific function of hCKα activity in PI4KIIIα-NS5A binding. This proposal is supported by the co-IP results in which hCKα activity facilitates the binding of PI4KIIIα to NS5A. Because hCKα shRNA reduces hCKα levels to approximately 13% of that detected in control stable knockdown cells, and CK37 does not completely attenuate hCKα activity, as reflected by the low levels of viral protein expression in CK37-treated, HCV-infected cells, residual hCKα protein and/or activity conceivably may still bridge PI4KIIIα and NS5A, resulting in incomplete abrogation of the NS5A-PI4KIIIα interaction.

We envision that the interfering effects exerted by CK37 on PI4P production and viral replication depend on the molar ratio of CK37 molecules to intracellular PI4KIIIα molecules in a defined context. Since the total level of overexpressed HA-PI4KIIIα and endogenous PI4KIIIα is approximately 4.3- to 4.7-fold that of endogenous PI4KIIIα expressed in cells lacking overexpression, unsurprisingly, functional HA-PI4KIIIα overexpression enables PI4KIIIα to outcompete CK37 molecules and “neutralize” the inhibitory effects of CK37, thereby restoring PI4P production, the ER translocation of PI4KIIIα and NS5A, and viral replication.

One possible mechanism by which hCKα activity modulates the PI4KIIIα-NS5A interaction is via modifications to the PI4KIIIα or NS5A structure and subsequent enhancement of the PI4KIIIα-NS5A interaction, resulting in PI4KIIIα activation in the ternary complex (Fig. 15B). This notion is supported by the finding that hCKα, in conjunction with NS5A, vigorously upregulates PI4KIIIα activity *in vitro* through hCKα activity. Therefore, the main function of hCKα during HCV replication is to recruit and closely bridge PI4KIIIα and NS5A, leading to the formation of a PI4KIIIα ternary complex. Then hCKα, in conjunction with NS5A, synergistically upregulates PI4KIIIα activity to facilitate abundant PI4P production and viral replication (Fig. 15B). Thus, the “trio” acts as a critical module to coordinately boost PI4KIIIα activation to achieve so as the massive PI4P production required to maintain the integrity of the MW structure and viral replication.

Because the translocation of hCKα, PI4KIIIα, and NS5A to the ER membrane is correlated with hCKα- and PI4KIIIα-stimulated PI4P generation, we propose that massive PI4P production drives the translocation of the hCKα-PI4KIIIα-NS5A ternary complex to viral replication sites on the ER membrane (Fig. 15B). This notion is supported by the findings that overexpression of wild-type (but not D288A mutant) hCKα-R upregulated the PI4P level and enhanced the ER transport of hCKα, PI4KIIIα, and NS5A in hCKα stable knockdown cells. The D288A single mutation in hCKα-R likely does not account for the impaired ER translocation of this mutant. In addition, the inhibited translocation of authentic PI4KIIIα and NS5A to the ER when the mutant hCKα-R was overexpressed was caused by the inability of this mutant to mediate elevated PI4P production. Because a single point mutation in the PI4KIIIα KD mutant likely does not drastically alter the ER translocation of this mutant, the redirection of PI4KIIIα and NS5A to the ER membrane via the overexpression of wild-type (but not KD mutant) PI4KIIIα in CK37-treated cells further supports a scenario in which mass PI4P synthesis occurs prior to PI4KIIIα and NS5A translocation to the ER (Fig. 15B).

Our previous (35) and present findings suggest that hCKα engages in multiple, complex protein-protein interactions with NS5A, PI4KIIIα, and NS5B during viral RC assembly on the ER membrane. The results obtained from the cell culture system and the PI4KIIIα *in vitro* activity assay presented here reveal the essential nature of the protein-protein interactions that lead to ternary complex formation to achieve robust PI4KIIIα activation. Further studies aimed at understanding the domains, motifs, and/or residues within hCKα, PI4KIIIα, and NS5A that are responsible for mediating the interactions between each set of paired counterparts and at characterizing JFH1 virus mutants harboring these mutations will not only elucidate the underlying molecular bases and allow us to further understand how protein-protein interactions impact the formation of the ternary complex but also reveal how hCKα acts as a key modulator to reshape basal and NS5A-stimulated PI4P production and promote viral replication.

Regarding the critical involvement of finely tuned PI4KIIIα activation in HCV replication (32), our study provides novel insight into the modulatory role of hCKα during HCV replication. hCKα functions as an indispensable, upstream coordinator of PI4KIIIα and NS5A by bridging them, reshaping their functions, and retargeting their intracellular localization to the PI4P-enriched, ER-derived membrane platform (Fig. 15B). On the PI4P-enriched membrane, the hCKα protein mediates the NS5A-NS5B interaction to assemble a functional RC (35), and PI4KIIIα activation continues to build the PI4P-enriched MW structure to harbor more viral RCs, leading to productive viral replication (Fig. 15B).

## MATERIALS AND METHODS

### Cells, antibodies, and plasmids.

The human hepatoma cell line Huh7 and its derivatives, Huh7 cells stably transduced with lentiviral vectors encoding control or hCKα shRNAs (control shRNA/Huh7 and hCKα shRNA/Huh7 cells, respectively) or with T7 RNA polymerase (T7/Huh7 cells), have been described previously (35). T7/Huh7 cells stably expressing lentivirally transduced control or hCKα shRNA (T7-control shRNA/Huh7 and T7-hCKα shRNA/Huh7 cells, respectively) have also been described previously (35). All cell culture-related reagents were obtained from Invitrogen.

The following antibodies were obtained commercially from the indicated vendors, and their catalog numbers are indicated in parentheses. The following mouse monoclonal antibodies (MAbs) were purchased: anti-Core (sc-57800; Santa Cruz), anti-NS3 (MAB8691; Merck Millipore), anti-NS5A (HCM-131-5; Austral Biologicals), anti-NS5B (HCV-4B8; BioFront Technologies), anti-Flag M2, anti-HA, and anti-β-actin (F1804, H3663, and A5441, respectively; Sigma-Aldrich), and anti-PI4P (isotype IgM) (Z-P004; Echelon). Mouse polyclonal normal IgG (12-371) was purchased from Merck Millipore. Rabbit polyclonal antibodies were purchased as follows: anti-hCKα (ab88053; Abcam), anti-Flag and anti-HA (F7425 and H6908; Sigma-Aldrich), and anti-PI4K (4902; Cell Signaling Technology). Goat antibodies were purchased as follows: anti-CALR and anti-HA (Y-11) (sc-6467 and sc-805-G; Santa Cruz). Anti-NS5A (MAb 9E10) was a gift from Charles M. Rice. Alexa Fluor-conjugated secondary antibodies were purchased from Invitrogen.

Previous reports have detailed the sources and/or construction of plasmids pUC-JFH1 (44); pWPI-T7-BLR, pTM-NS3–5B, pTM-NS5A, and pTM-HA-PI4KIIIα (24, 45); pEF1A-HA-PI4KIIIα-WT and pEF1A-HA-PI4KIIIα-KD (28); and pCMV6-hCKα, pCMV6-hCKα-R, and pCMV6-hCKα-D288A-R (35).

### Reagents and siRNAs.

CK37 (CAS1001478-90-5) was purchased from Merck Millipore, and 3-[(3-cholamidopropyl)dimethylammonio]-1-propanesulfonate (CHAPS; catalog no. 4145-00) was obtained from J. T. Baker. Cycloheximide (C104450), dimethyl sulfoxide (DMSO; D8418), and PAO (P3075) were obtained from Sigma-Aldrich. The following recombinant purified proteins were obtained: PI4KIIIα (PV5689; Thermo Fisher Scientific), NS5A (LS-G18066; LifeSpan BioSciences, Inc.), and hCKα (TP307209; Origene). An ADP-Glo kinase assay kit (V9101) and a CellTiter-Glo luminescent cell viability assay kit (G7571) were purchased from Promega. Phosphatidylinositol-phosphoserine (PI:PS; PV5122) was obtained from Invitrogen. AL-9 was a gift from Raffaele De Franscesco. siRNAs targeting hCKα (HSS140690 and HSS140691) and PI4KIIIα (HSS108026, HSS108027, and HSS182310) were purchased from Invitrogen. Nontargeting control siRNA (29551) was obtained from Santa Cruz.

### HCV production and infection.

JFH1 RNA was generated by *in vitro* RNA transcription and electroporated into Huh7 cells to produce cell culture-derived infectious HCV (HCVcc). The titration and infection of HCVcc at the multiplicity of infection (MOI) of 1 were performed based on a previously described procedure (35). Cells infected with HCVcc were harvested 72 h postinfection for Western blot analysis or confocal microscopy.

### Plasmid DNA transfection.

For Western blot analysis, confocal microscopy, and PI4P measurements, 2 × 10^5^ Huh7, T7/Huh7, T7-control shRNA/Huh7, or T7-hCKα shRNA/Huh7 cells were seeded in 6-well plates overnight and were then transfected with 4 μg of each plasmid using Lipofectamine 2000 based on a previously described procedure (35). For co-IP analyses, 1 × 10^6^ T7/Huh7, T7-control shRNA/Huh7, or T7-hCKα shRNA/Huh7 cells were seeded in 6-cm plates overnight and were then transfected with 8 μg of each plasmid examined. The appropriate vectors were added to the transfection reactions, and the total DNA amounts in all transfections in a set were identical. The cells were harvested 48 h posttransfection and were lysed with phosphate-buffered saline (PBS) (pH 7.4) containing 1% NP-40, 1% sodium deoxycholate, and a protease inhibitor cocktail for the Western blot analyses or with Tris-HCl (pH 7.4) containing 150 mM NaCl, 1% CHAPS, and a protease inhibitor cocktail for co-IP analysis. Alternatively, the transfected cells were examined by confocal microscopy for the intracellular localization of the proteins or the assessment of PI4P levels. In certain cases, the siRNA-transfected cells were transfected with the plasmids examined. Additionally, the infected cells were transfected with the plasmids and then subjected to inhibitor treatment studies.

### siRNA silencing and inhibitor studies and cell viability assay.

For the RNAi silencing studies, the cells grown in 6-well plates were transfected with 100 pmol of siRNAs using Lipofectamine RNAiMax (Invitrogen) according to the manufacturer's protocol. At 24 h post-siRNA transfection, the cells were left uninfected or infected HCVcc and were then subjected to a Western blot analysis. Alternatively, the siRNA-transfected cells were transfected with plasmid DNAs prior to Western blotting or co-IP analysis.

For the inhibitor studies, the cells seeded in 6-well plates were infected with or without HCVcc. The inhibitors CK37 and AL-9 were prepared in DMSO and were added to cultured cells at final concentrations of 100 μM and 5 μM, respectively, at 48 h post-HCVcc infection or 24 h post-DNA transfection. The cells treated with DMSO (vehicle) at the same concentration as that in the inhibitor treatment were used as controls. The cells were harvested 24 h after inhibitor treatment for Western blot analysis or confocal microscopy. Alternatively, the transfected cells were treated with CK37 or PAO at a final concentration of 100 μM or 5 μM and were subjected to co-IP analysis. The viability of the HCV-infected cells treated with a vehicle or different inhibitors was ascertained using a CellTiter-Glo luminescent cell viability assay kit.

### Cycloheximide chases.

For the cycloheximide chases, the Huh7 cells grown in 6-well plates were transfected with the control or hCKα siRNA. At 48 h after siRNA transfection, the cells were treated with cycloheximide at a final concentration of 100 μg/ml and were then collected at different time points post-cycloheximide addition for Western blot analysis. The percentage of PI4KIIIα detected at different times relative to that detected at time zero in each siRNA transfection, which was arbitrarily assigned a value of 100%, was expressed.

### SDS-PAGE, immunoblotting, and co-IP analyses.

SDS-PAGE, Western blotting, and immunoblot visualization were performed as indicated previously (35). The levels of the proteins indicated in the immunoblots were quantified using ImageQuant TL software (GE Healthcare), and the relative protein levels were determined. For co-IP analysis, 1 μg of the antibody was first incubated with protein A Mag Sepharose beads (28-9670-62; GE Healthcare) at 4°C for 2 h, followed by incubation with cell lysates at 4°C for 6 h. After the beads were washed 5 times in PBS, they were boiled with sample loading buffer and were subjected to SDS-PAGE and immunoblot analyses.

### Confocal laser scanning microscopy and PI4P quantification.

For the confocal microscopy analysis and quantification of the colocalization of the proteins, the HCV-infected or DNA-transfected cells were fixed, immunostained with specific antibodies, and examined by confocal microscopy based on previously described procedures (35). The images were edited using Zen 2011 software. The colocalization of the indicated proteins was quantified using ImageJ or MBF ImageJ from 20 randomly selected cells as previously described (35).

For the PI4P quantification, the cells were prepared as described previously (24). Z stack images of cells were acquired with a Zeiss LSM 780 confocal laser scanning microscope using a 40× objective lens. Then the z projections of the z stack image were generated with the “sum slices” option in ImageJ, followed by thresholding the signal intensity of PI4P staining. The PI4P amount in 300 cells was determined by defining the cell area, and the integrated density (IntDen) value of the PI4P signal in each cell was obtained by the “analyze particles” function in ImageJ.

### *In vitro* PI4KIIIα assay.

PI4KIIIα *in vitro* activity was determined with 30 ng of purified recombinant PI4KIIIα in the presence or absence of 30 ng each of purified recombinant NS5A and/or hCKα according to the method previously described by Bianco et al. (41) using an ADP-Glo kinase assay kit. Bovine serum albumin was added into the kinase reaction mixture so that all reaction mixtures contained the same total amounts of protein. Reactions were also performed without the PI:PS substrate to detect contaminating ATPase activity present in the test conditions. This activity was subtracted from the measured kinase activity. Kinase activity was expressed as the fold change in the luciferase activity under test conditions relative to that detected in the presence of PI4KIIIα alone, which was arbitrarily assigned a value of 1.

### Data and statistical significance analyses.

The results from the Western blot analysis and the relative protein quantification in the immunoblots were obtained from three independent studies. The Western blot data for representative sets are shown in the figures. All data are presented as the means ± standard deviations (SD), and statistical analyses were performed using a two-tailed, unpaired Student *t* test. Differences between the two indicated settings were considered statistically significant at a *P* value of <0.05.
